# Innovative IgG Biomarkers Based on Phage Display Microbial Amyloid Mimotope for State and Stage Diagnosis in Alzheimer’s Disease

**DOI:** 10.1021/acschemneuro.9b00549

**Published:** 2020-03-16

**Authors:** Laura M. De Plano, Santina Carnazza, Domenico Franco, Maria Giovanna Rizzo, Sabrina Conoci, Salvatore Petralia, Alessandra Nicoletti, Mario Zappia, Michela Campolo, Emanuela Esposito, Salvatore Cuzzocrea, Salvatore P. P. Guglielmino

**Affiliations:** † Department of Chemical, Biological, Pharmaceutical and Environmental Sciences, University of Messina, Viale F. Stagno d’Alcontres 31, 98166 Messina, Italy; ‡ STmicroelectronics, Stradale Primosole, 50, 95121 Catania, Italy; § Distretto Tecnologico Micro e Nano Sistemi Sicilia, Strada VII-Zona Industriale, 95121 Catania, Italy; ∥ Neurology Clinic, Department “G.F. Ingrassia”, Section of Neurosciences, University of Catania, Via Santa Sofia 78, 95123 Catania, Italy

**Keywords:** Phage display double binding selection, amyloid conformational epitopes, Aβ_1−42_ cytotoxicity inhibition, fibril disaggregation, autoantibody detection, AD state and stage progression screening

## Abstract

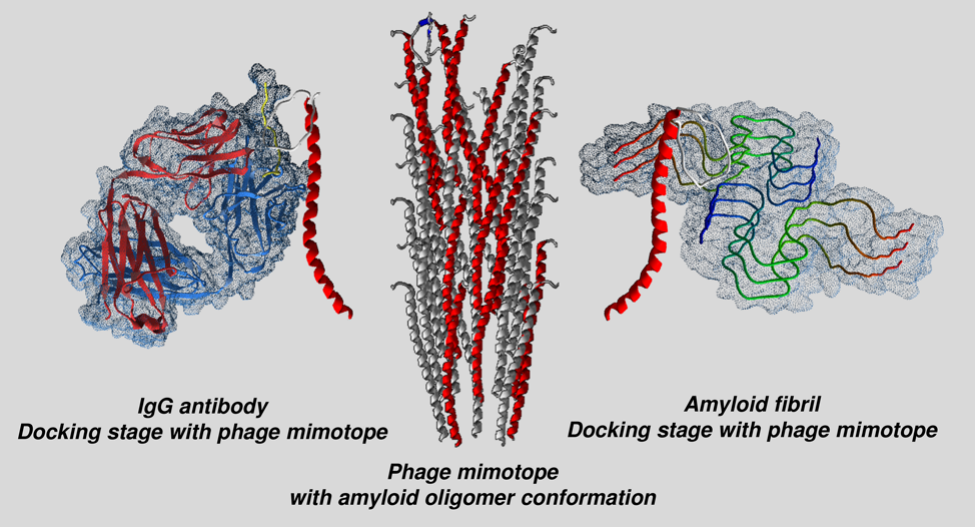

An innovative approach to identify new conformational antigens of Aβ_1–42_ recognized by IgG autoantibodies as biomarkers of state and stage in Alzheimer’s disease (AD) patients is described. In particular, through the use of bioinformatics modeling, conformational similarities between several Aβ_1–42_ forms and other amyloid-like proteins with F1 capsular antigen (Caf1) of *Yersinia pestis* were first found. pVIII M13 phage display libraries were then screened against YPF19, anti-Caf1 monoclonal antibody, and IgGs of AD patients, in alternate biopanning cycles of a so-called “double binding” selection. From the selected phage clones, one, termed 12III1, was found to be able to prevent *in vitro* Aβ_1–42_-induced cytotoxicity in SH-SY5Y cells, as well as to promote disaggregation of preformed fibrils, to a greater extent with respect to wild-type phage (pC89). IgG levels detected by 12III1 provided a significant level of discrimination between diseased and nondemented subjects, as well as a good correlation with the state progression of the disease. These results give significant impact in AD state and stage diagnosis, paving the way for the development not only for an innovative blood diagnostic assay for AD precise diagnosis, progressive clinical assessment, and screening but also for new effective treatments.

## Introduction

Alzheimer’s disease (AD) is the most common form of dementia and is a leading cause of morbidity and mortality worldwide. In the population of most industrialized countries, AD cases are rapidly increasing with the aging society; thus there is a crucial need for new methods for early diagnosis and prevention. Diagnostic guidelines for Alzheimer disease include three stages: preclinical, mild cognitive impairment (MCI), and dementia. Cognitive impairments are considered the first appearing symptoms and signs of the disease. Currently, the biological label of AD disease is given if the person has demonstrated positive biomarkers for both amyloid and pathologic tau. The preliminary diagnosis of AD is made by a combination of laboratory and clinical criteria, which include neuropsychiatric tests, behavioral history assessments, and neuroimaging techniques.^1−3^ In addition, more recent clinical studies have demonstrated biomarkers of “suspected non-Alzheimer disease pathophysiology” (SNAP) causing amnestic type cognitive impairment.^4^ Also other proteinopathies have been associated with substantial cognitive impairment that mimics AD clinical syndrome (i.e., limbic-predominant age-related TDP-43 encephalopathy, LATE).^5^

In any case, since changes in the brain precede the appearance of the disease by about a decade, scientific research is increasingly oriented to the discovery of new biomarkers that act as indicators of the beginning of the pathological process and its progression. To date, there are no predictive diagnostic systems capable of monitoring the disease.

Many studies have been focused on biomarker discovery in a noninvasive way in different body fluids, such as cerebrospinal fluid (CSF), saliva, urine, and blood.^6−8^ Serum levels of antibodies specifically binding Aβ (Aβ autoantibodies) in AD and in non-AD control subjects have been extensively investigated as potential blood biomarkers applied to AD diagnosis.^9−12^ However, the results of these studies are contradictory. In fact, some authors reported high levels of anti-Aβ IgGs in subjects with AD compared to healthy controls,^13^ whereas others showed a reduction of anti-Aβ IgGs in AD patients^14−16^ or even no difference.^17,18^

These results appeared inconsistent due to several factors affecting the detection of specific IgGs against Aβ_1–42_, including nonspecific binding,^19^ low avidity and low levels in serum,^20^ incorrect diagnosis,^21^ circulation of Aβ autoantibodies both in free and in antigen-bound form,^22−24^ and, not least, the structural conformation of Aβ_1–42_.^25^

The structural plasticity of amyloid is partially based on its polymorphism, the ability to form aggregates of different structures;^26−28^ indeed, Aβ fibrils are polymorphic mainly due to conformational differences in the folding of their unifying structural feature, which is their specific type of β-sheets.^29−32^

Amyloid structures are usually divided into two main groups based on the presence of parallel in-register versus antiparallel β-sheet structures. However, it is progressively more evident that within each group there is considerable structural diversity. These polymorphisms are self-propagating and give rise to differences in the quaternary structure of the fibrils, which differ significantly from native, monomeric protein folding for conformation and set of inter-residue interactions.^33^ In this condition, folding polymorphisms assume higher order structure, and the amino acids involved in the reactive domain of ligands are often dispersed in the protein primary sequence.

On the other hand, conformation-dependent antibodies have been reported recognizing a generic epitope specific to many types of amyloid fibrils regardless of their amino acid sequences, as they also bind to other disease-related amyloid fibrils and amyloid-like aggregates derived from other proteins of unrelated sequence.^34−36^ These antibodies do not bind to the native amyloid protein precursors and not even to other kinds of protein aggregates, indicating that fibrils display a distinct conformation-dependent, sequence-independent epitope that is absent in prefibrillar oligomers.^25,36,37^

It is interesting to observe also that a group of natively folded proteins are immunopositive to the aforesaid conformation-dependent antibodies, suggesting that they share a common structure, the so-called amyloid oligomer conformation.^38^ In fact, similar amyloid oligomer conformations were found in several bacterial, yeast, human, and mammalian proteins.

More recently, Hatami et al.^39^ characterized the immune response to fibrillar Aβ_1–42_ in rabbits, demonstrating that it reflects the diversity in amyloid structures. They isolated 23 OC-type monoclonal antibodies (mOCs) recognizing different epitopes associated with several polymorphic structural variants of Aβ_1–42_ fibrils. OC is a rabbit polyclonal antiserum that binds to fibrillar Aβ, as well as other similar amyloid-like protein structures but not to monomers or prefibrillar oligomers.^36^ These authors also observed that many epitopes of mOCs were discontinuous and in any case not sequence-specific but conformation-dependent; in fact, even those recognizing a linear segment of Aβ reacted also with amyloid fibrils from unrelated sequences. This suggests that reactivity with linear segments may reflect the tendency of short peptides to form amyloid-like structures with generic fibril epitopes rather than a specificity for the particular sequence. These antibodies remarkably showed different patterns of immunoreactivity in AD and transgenic mouse brain, in which they identified morphologically and spatially distinct types of amyloid deposits.

It is known that antibody ligands with structures partly or completely unlike the native antigen can be isolated by library screening. In this view, peptide libraries have been successfully screened for “conformational mimotopes” that bind to carbohydrate-specific antibodies, suggesting the use of these selected peptides as vaccines raising antibody levels against the native carbohydrate antigen.^40−42^ Shin et al.^41^ have identified, by mAb-biopanned phage display libraries, a peptide able to assume various conformations and mimic pneumococcal as well as meningococcal capsular polysaccharide, binding more than one unrelated antibody. More specifically, computerized analysis with phage display technology was used to identify conformational mimotopes of putative self-antigens in the circulating IgGs present in the serum of AD patients.^43^ More recently, an approach combining phage display and protein microarrays was used to identify a panel of peptide targets of AD autoantibodies.^44^ Among the most immunoreactive sequences identified, only one was identical to the amino acid sequence of a known protein, whereas the others were mimotopes, which are peptides with no linear sequence identity but mimicking the structure of conformational B-cell epitopes with immunogenic properties and diagnostic ability.^44^

It is reasonable to assume that the deposition of amyloids with diverse misfolding can self-assemble and generate new epitopes (e.g., discontinuous epitopes derived from amyloid-based aggregation) that could induce an immune response.

Since the conformational epitopes are exposed only in some aggregation states of proteins, the search for antibodies to these types of antigens is very difficult. Despite this, it could be very useful to detect the presence of antibodies against conformational epitopes of the aggregation states of amyloid fibrils in order to define the state and stage markers of the disease.

In the present study, we explore the possibility to detect in sera of AD patients IgGs that recognize “conformational epitopes” of Aβ fibrils formed during the progression of Alzheimer’s disease, through the identification of conformational structures similar to fibrillar Aβ_1–42_.

Our approach does not aim to directly identify the native antigen of specific AD antibodies or a mimotope thereof but rather to search for a common conformational antigen selectively recognized by circulating antibodies in AD patients.

To achieve this, we exploited structural peptide segments able to form amyloid-like structures “common” to Aβ fibrils and other unrelated proteins as target for serum circulating IgGs of patients with AD, through the use of a “double binding” biopanning screening of pVIII M13 phage display libraries, looking for a “generic conformational antigen” to employ in a diagnostic system. To this aim, structural similarity search was first performed with bioinformatics tools among several Aβ_1–42_ misfolded structures and other proteins properly designed to have the structural bases for conformational similarity. Among analyzed proteins, excluding those present in human and common infectious agents, *Yersinia pestis* F1 capsular antigen (Caf1) was chosen for the significant structural homology found between its epitopic regions and the fibrillar form of β-amyloid. Thus, phage display M13 libraries were first selected against both the mAb YPF19 recognizing Caf1 antigen and then the serum samples of AD patients used as baits in alternate biopanning cycles of the so-called double binding phage display selection. Among the most reactive selected phage pool, one 12-mer random peptide phage clone, termed 12III1, was found to significantly enhance inhibition *in vitro* of Aβ fibrillation as well as disaggregation of preformed fibrils, compared to pC89 wild-type vector without peptide. Therefore, this phage was used in ELISA assay to detect serum IgGs in patients with AD and in healthy control subjects. Theranostic results are herein presented and discussed.

## Results and Discussion

AD is today the main cause of dementia in the population, and it is associated with misfolding and aggregation states of β-amyloid. The aggregation states of this peptide are correlated with the onset and progression of disease. β-Amyloid forms insoluble aggregation structures, extracellular plaques, and soluble oligomers. Both extracellular plaques and oligomers are thought to be responsible for the brain degeneration observed in patients with AD.

There have been several important studies suggesting significant structural polymorphism among fibrillar amyloid aggregates.^29,31,45,46^ The aggregation states of β-amyloid are similar to those observed in different amyloidogenic proteins able to form polymorphic structural variants, characterized by the formation of hydrogen bonds determining β-sheet aggregates, including parallel, in-register β-sheets and antiparallel β-sheets, β-solenoids, β-barrels, and β-cylindrins.

Antibodies against amyloid-forming peptides and aggregates have attracted broad interest for their utility as potential therapeutic agents and research tools.

As mentioned in the Introduction, polyclonal sera to Aβ oligomers and fibrils are found in rabbits that are highly conformation-dependent, generic sequence-independent.^39^ Conformation-dependent antibodies have been reported recognizing generic epitopes specific to many types of amyloid fibrils with little apparent dependence on amino acid sequence.^34^

The amyloid oligomer represents a generic conformation common to protein structure of different organisms, such as human, bacteria, yeast, and animals. In particular, using an anti-amyloid oligomer conformation-specific antibody, it has been proven that the native fold of some chaperone and nonchaperone proteins exhibits the oligomer conformation and could be correlated with anti-β-aggregation.^37,38^

On the other hand, it could be clinically very relevant to detect in Alzheimer’s disease patients the presence of antibodies against conformational epitopes of the amyloid fibril aggregation states since this will enable identification of markers for the state and stage of the disease.

### Bioinformatics Analyses

To achieve the indirect approach for detection of IgG antibodies in sera of AD patients herein proposed, based on the recognition of generic conformational epitopes of amyloid fibrils correlated to AD-state or stage, specific bioinformatics analysis was performed.

To this purpose, a conformational mimotope in unrelated amyloidogenic proteins was searched and identified, able to be recognized both by IgG antibodies present in serum from AD patients (AD sera) and by the monoclonal antibody specific for the target sequence in an unrelated protein.

The bioinformatics approach allows us to obtain information about the amyloid oligomer conformation present in proteins and molecules.^43,47,48^

Template proteins were searched among proteins from different species, using UniProtKB tool as described in Methods, and 47 proteins were identified (TableS1 in Supporting Information).

Proteins uncharacterized or without identified 3D structure or belonging to infectious agents involved in common human diseases or commensal bacteria of the human microbiome were manually excluded.

Similarity was observed between Caf1 of *Yersinia pestis* and fibrillar Aβ_1–42_. F1 antigen (UNIPROT ID P26948) was chosen as target of analysis in 3D alignment to verify the conformational match with Aβ structures.

Caf1 includes linear fibers of monomers subunits assembled via the chaperone/usher pathway.^49,50^ In particular, Caf1 is a β-structural protein that in the polymeric form has very high conformational stability. The 2.2 Å resolution crystal structure of the ternary Caf1M/Caf1/Caf1N complex revealed that Caf1 is an incomplete β-sandwich immunoglobulin-like fold.^51^ The final stable fold is the result of replacement of the chaperone parallel β-strand by the “spare” antiparallel β-strand from the N terminus of the subsequent subunit, thus linking them to form a chain. Similarly, it is known that the 3D structure of the Aβ_1–42_ protofilament is formed by two stacked, intermolecular, parallel, in-register β-sheets that perpetuate along the fibril axis.^30^ A recent study by cryoelectron microscopy to 4-Å resolution, complemented by solid-state nuclear magnetic resonance experiments, showed that the β strands are staggered with relation to one another in a zipper-like manner.^52^

On the other hand, it is well-known that Aβ exists in several polymorphs with varying width and helical pitch, different cross-section profiles, and different interactions between the monomers.^52^

**Figure 1:**
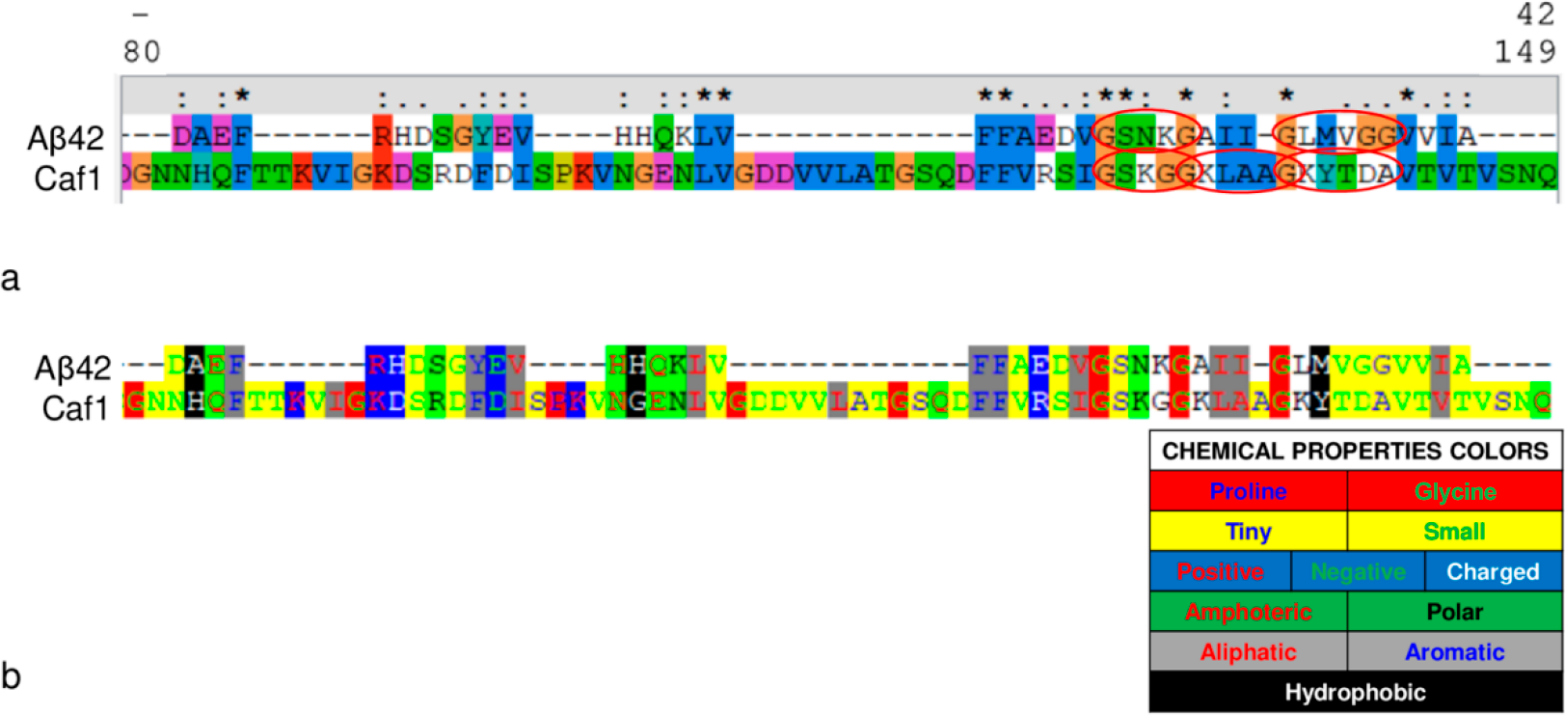
Sequence alignment between Aβ_1–42_ and Caf1 by Clustal X2.1 (a) and Genedoc (b). GxxxG, GxxxxG, and GxxxxA motifs are circled in red. Physiochemical properties of amino acids involved in alignment are shown in panel b, and Genedoc color legend is reported.

The linear alignment (Figure 1) essentially concerns the following Aβ_1–42_ regions: the region Aβ(1–16), epitopic for immunoglobulins and involved in the formation of protofilaments,^53,54^ and the region Aβ(21–37), recognized by “fibril-inhibiting” Aβ autoantibodies.^55,56^ Moreover, it is known that Aβ(4–10) is epitopic for “plaque-specific” antibodies inhibiting both Aβ fibrillogenesis and cytotoxicity.^57,58^ On Caf1, the regions mainly involved in alignment (Figure 1 fall within two B (84–101; 121–144) and one T (102–116) cell epitopes.^59^ In particular, F1(121–144) was demonstrated as one of the immunodominant and highly immunogenic sequences of F1 protein, which is recognized by a high amount of antibodies raised against the native F1 antigen.^60^

Conformational similarity analyses were then conducted, based on identifying residues occupying an equivalent geometric shape in space. The match between the structures was observed in amino acids distant in the primary sequence but contiguous in the 3D structure (see section 1 of Supplemental Data in Supporting Information for more details). In addition, RCSB Sequence & Structure Alignment tool was used in order to perform the 3D alignment of fibrillar amyloid (PDB ID 2nao) with the single specific chains of F1 antigen (identified by PDB IDs 1p5u, 1z9s, 3dos, 3dpb, and 3dsn). As already evoked by the primary sequence alignment, amino acid regions of Aβ_1–42_ involved in 3D alignment are the following: 18-VFFAEDVGSNKGAIIGLM-35 (with 1p5u.C, 3dsn.C), 9-GYEVHHQKLVFFAE-22 (with 1z9s.C, 3dpb.C), and 1-DAEF--------RHDSGYEVHHQKLVFFAE-22 (with 3dos.C), comprising the main Aβ epitopes already discussed above. Intriguingly, on Caf1, in all the PDB structures analyzed, conformational alignment with Aβ_1–42_ fibril falls within the immunodominant region (from 121-F to 143-T). See section 2 of Supplemental Data in Supporting Information for more details. The conformational homology found between the different forms taken by Caf1 and Aβ_1–42_ fibril confirms the lack of probability that these are randomly similar. Having found a sufficient degree of conformational similarity between Aβ_1–42_ in fibrillar form and F1 antigen implies that the latter protein includes some structural conformations resembling the Aβ_1–42_ protein. In particular, the sequence overlap between the C-terminal of β amyloid and the immunodominant region of Caf1 observed in linear alignment (50% similarity, 29% identity) concerned the GxxxG, GxxxxG, and GxxxxA repeating motifs^61^ (Figure 1a) and the small amino acid arrangement (Figure 1b), which conferred the particular conformation to the proteins and were recently demonstrated as being primarily responsible for both Αβ self-assembly and neurotoxicity.^62^

On the basis of bioinformatics analysis, we consider F1 as a protein with amyloid oligomer conformation like Aβ_1–42_ protein.

### Double Binding Phage Display Selection

In order to identify a hypothetical common conformational epitope between F1 and misfolded AD-specific Aβ_1–42_ proteins, M13 phage display libraries were used in a double binding selection in alternate biopanning with mAb YPF19 (the monoclonal antibody for F1 antigen) and a pool of five AD sera IgGs (see Methods). Antibody-associated phage isolated by acid elution in each biopanning cycle were separately amplified in *E. coli* strain TG1 and used for subsequent rounds of selection. After each round of selection, the yield of phage eluted from the Dynabeads–antibodies was determined prior amplification (Table 1).

**Table 1:** Yields of “Double-Binding” Selection of M13 pVIII-12aa and pVIII-12aa-Cys Phage Display Libraries

	12aa-Cys			12aa		
	in^a^	out^b^	yield^c^	in^a^	out^b^	yield^c^
round I (against mAbYPF19)	3.5 × 10^12^	3.3 × 10^7^	9.43 × 10^–6^	1.5 × 10^12^	1.3 × 10^7^	8.67 × 10^–6^
round II (against AD IgGs)	1.45 × 10^13^	4.2 × 10^9^	2.9 × 10^–4^	1.7 × 10^13^	2.65 × 10^9^	1.56 × 10^–4^
round III (against mAbYPF19)	3.6 × 10^12^	3.65 × 10^8^	1.01 × 10^–4^	5.32 × 10^12^	8.25 × 10^8^	1.55 × 10^–4^
round IV (against AD IgGs)	8.75 × 10^12^	2.75 × 10^9^	3.14 × 10^–4^	1.8 × 10^12^	1.5 × 10^9^	8.33 × 10^–4^

a Input quantity of engineered bacteriophage at the start of the biopanning process.

b Quantity of phage recovered at the end of each selection round.

c Input to output ratio to indicate the number of bacteriophage remaining bound to the selection target.

### Immunoscreening and ELISA Assays Using AD Sera

Phage clones selected from the eluted phage population of each library, being the most reactive in immunoscreening, were independently propagated and tested in indirect ELISA assays using both mAb YPF19 and AD sera. Obtained results showed that three 12aa and four 12aa-Cys clones were significantly reactive (data not shown), suggesting that they displayed peptides likely miming conformational epitopes recognized by circulating IgGs from AD patients but also by the monoclonal antibody specific for Caf1.

**Figure 2:**
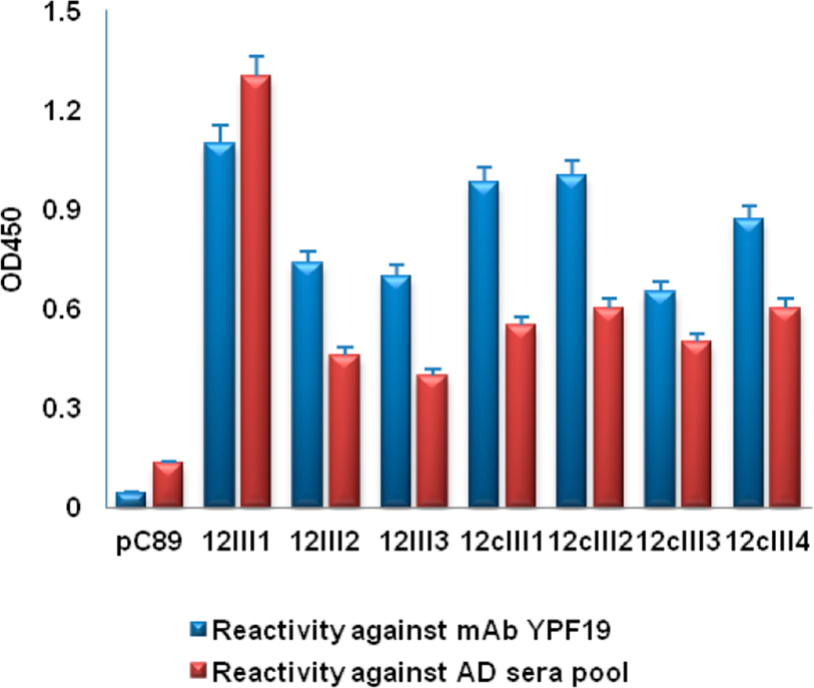
ELISA reactivity of phage clones isolated by double binding selection against mAb YPF19 and pool of AD sera.

The phage clones 12III1, 12cIII1, 12cIII2, and 12cIII4 were the most reactive, whereas pC89 wild-type vector showed very low reactivity against both mAb YPF19 and human sera (Figure 2.

### Sequence Analysis of Selected Phage Clones

Individual phage clones were independently propagated for further analysis and their DNA sequenced to determine the peptide sequence displayed in their major coat protein. Their sequences are showed in Table 2.

**Table 2:** Amino Acid Sequences of Isolated M13 pVIII-12aa and pVIII-12aa-Cys Phage Clones

phage clones	sequence
12III1	RWPPHFEWHFDD
12III2-3	WEYDRYRGWHIG
12cIII1-2	GGGCIEGPCLEG
12cIII3	WVGCHGEWCGVW
12cIII4	HRGCIEGPCLDA

Sequence exposed on phage clones 12III2 and 12III3 was the same, as well as that on clones 12cIII1 and 12cIII2 and partially on 12cIII4. In contrast, phage clones 12III1 and 12cIII3 showed a single unrelated sequence. Finally, 12III1 showed major reactivity for both mAb-YPF19 and pool of AD sera.

Since peptide clusters with similarity were not found in the linear peptide exposed in the unconstrained library, it was not possible to identify a consensus sequence or common sequence motifs with the amino acid sequence of F1 antigen or Aβ_1–42_. The selected phage clones did not show any amino acid sequence similar to the phage clone previously identified by using a phage–peptide library of filamentous phage displaying random combinatorial peptides,^53,54^ bearing the epitope EFRH, corresponding to the first amino acids of the human β-amyloid, that controls as a regulatory site both the formation and disaggregation process of Aβ fibrils.

In our study, only data obtained with the most reactive 12III1 clone, selected from M13 pVIII-12aa library, are reported, since it preferentially interacted with the selection targets (pool of AD sera and mAb-YPF19). In particular, the linear sequence of 12III1 phage clone displayed peptide aligned along discontinuous regions of both Αβ42 and Caf1 protein, so it was not possible to identify a common sequence (See section 3.1 of Supplemental Data in Supporting Information). This reflects the epitope being often composed of discontinuous residues onto the linear sequence of the antigens, and hence, antibody–antigen interactions are mediated through tertiary structure. In this case, 12III1 would act as a conformational mimotope. To verify this hypothesis, the three-dimensional alignment was performed. By using Mapitope algorithm, as well as combined Pepsurf and Mapitope algorithms, the best 3D alignment cluster for 12III1 peptide on Aβ fibril was 3-EFRH-6, and those for peptide flanked by the exposed amino acids of pVIII protein were 20-FAEDVG-25 and again 3-EFRH-6. On the other hand, based on the results obtained with the above-mentioned algorithms, 12III1 aligned on F1 protein mainly with 74-F 82-HQF-84 region and within the immunodominant 126-IGSKGGKLAAGKYTDAVT-143 region (See section 3.2 of Supplemental Data in Supporting Information for more details).

It is noteworthy that the amino acids involved in the match fall within the sequences involved in the initial three-dimensional alignment between Aβ and microbial amyloid F1, mainly included in the epitopic regions of both proteins.

### 12III1 Phage Clone Ability to Discriminate Pool Sera of AD from Healthy Individuals (CTR)

In order to evaluate whether IgG serum levels against 12III1 phage clone were able to discriminate the immune response between AD patients and healthy subjects, this clone was tested in ELISA assay against a pool of five human sera from AD subjects and a pool of five human sera from healthy individuals (CTR). Results obtained showed a mean OD_450_ value of 1.266 against AD sera and a mean of 0.464 against healthy sera (*p*-value < 0.0001), demonstrating the presence of serum IgG levels toward the displayed peptide that were significantly higher in AD patients.

### Enhancement of 12III1 Phage Inhibition of Αβ42 Cytotoxicity in Vitro

It is known that filamentous bacteriophage M13 can mediate disruption of amyloid assemblies;^63^ in particular, Krishnan et al.^64^ showed that highly purified preparations of native M13 widely and strongly mediate binding to and disruption of various misfolded protein assemblies, including Aβ, α-synuclein, tau, and yeast prion Sup35.

**Figure 3:**
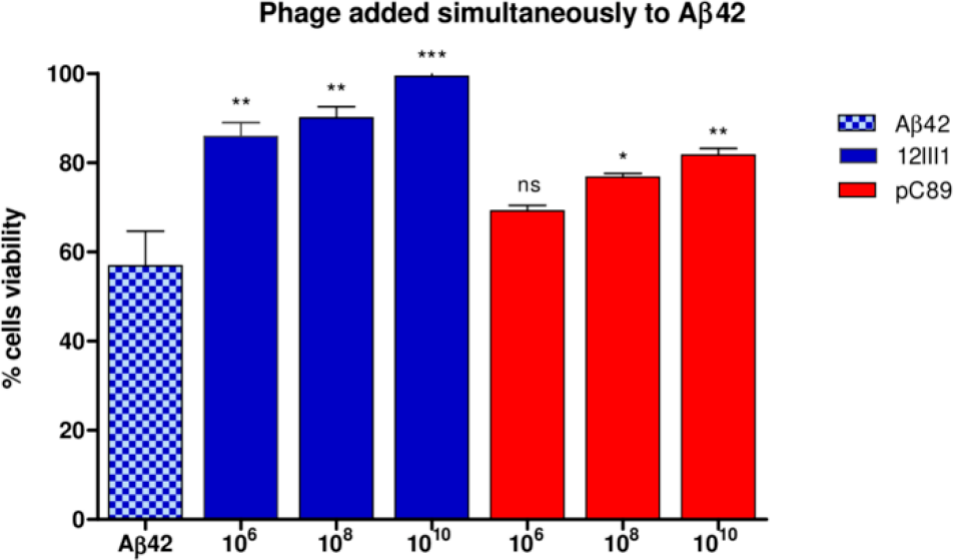
Inhibition of Aβ_1–42_-induced cytotoxicity at increasing concentrations of 12III1 recombinant phage clone and pC89 wild-type phage, as evaluated in SHSY-5Y cell viability *in vitro* test.

Fibrillation implies self-aggregation of β monomers, and the phage, being a conformational mimotope, could interact with β-amyloid. *In silico* molecular docking modeling provided a prediction of the 3D molecular interaction between Αβ_1–42_ fibrils and engineered 12III1–pVIII protein through amino acid residues of the displayed peptide (see section 3.3 of Supplemental Data in Supporting Information for more details). In order to evaluate the effect of 12III1 exposed peptide on disassembling amyloid fibrils compared to M13 phage (pC89), we tested *in vitro* its ability to interact with Αβ_1–42_. We first used 12III1 and pC89 phage clones as inhibitors of Aβ aggregation, in SHSY-5Y cell viability *in vitro* test. Phage were used at different concentrations (phage titer 1 × 10^6^, 1 × 10^8^, and 1 × 10^10^). Aβ_1–42_ showed cytotoxicity of about 60%; phage clones by themselves showed no cytotoxicity *in vitro* on SHSY-5Y cells at any time tested (data not shown). Both 12III1 and pC89 wild-type phage showed significant inhibition of Aβ_1–42_-induced cytotoxicity when added to cell cultures simultaneously with Aβ_1–42_. However, pC89 wild-type phage exhibited a lower inhibition of Aβ_1–42_-induced cytotoxicity than 12III1 phage at all the assayed titers (Figure 3).

**Figure 4:**
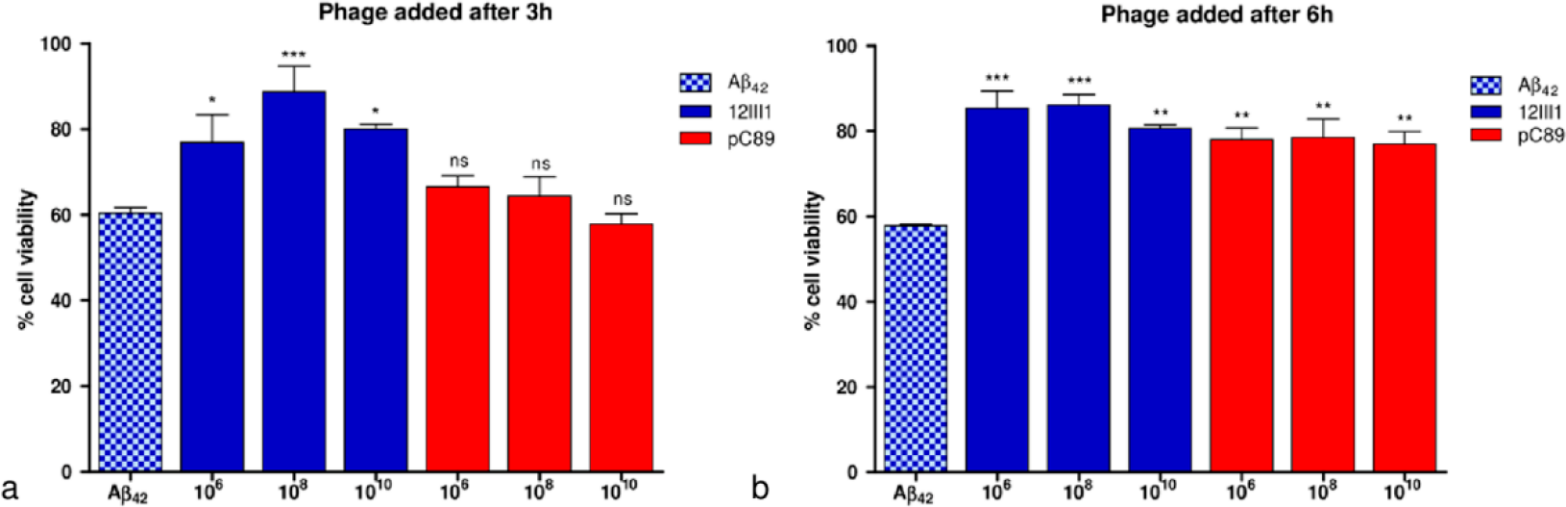
Reduction of Aβ-amyloid cytotoxicity in SHSY-5Y cell viability *in vitro* test after addition of pC89 wild-type phage and 12III1 recombinant phage clone, at 3 h (a) and 6 h (b) following Aβ-stimulation.

Moreover, when phage were added after 3 and 6 h following Aβ stimulation, 12III1 phage restored cell viability (% comparable to control cells with no Aβ and no phage), and pC89 wild-type phage showed no activity at any tested titer after 3 h, whereas increased cell survival was observed after 6 h but to a lower extent than with 12III1 (Figure 4). Data suggest that 12III1 displayed peptide increases affinity to Aβ fibrils, promoting disassembling of toxic preformed Aβ aggregates.

It is already known that pIII M13 phage coat protein mediates a generic binding to the amyloid fold, predominantly through middle and C-terminal residues of the Aβ subunit by β strand interactions.^64^ Since three different amyloid fibers resulted as targets for M13-mediated binding and remodeling, it was suggested that the interaction with the phage is conformation and not protein primary sequence dependent. The bacteriophage minor capsid protein pIII was proven to be critical for this activity, inducing binding and disruption of amyloids by its two N-terminal domains as a general amyloid interaction motif (GAIM). pIII uses TolA-C binding sequences (including the pIII β4 strand) to recognize and bind the canonical amyloid fold represented in Aβ fibers.^63^ This is the case for pC89 phage vector inhibitory capacity of Aβ cytotoxicity observed in this study against human SH-SY5Y cells.

However, the 12III1 clone exposed peptide, inserted in-frame in the major pVIII coat protein, increased cell survival to a much greater extent than the wild-type phage (with no insert), even restoring cell viability (Figure 3). Indeed, this specific phage clone was able to prevent Aβ_1–42_ amyloid fibrillation, as well as to promote disaggregation of preformed fibrils, more effectively than pC89 (Figure 4). These results suggest that the recombinant peptide displayed on pVIII coat protein could give the phage the ability to interact with Aβ fibrils in a different way than pIII coat protein, probably by virtue of conformation assumed. In any case, 12III1 showed a significant enhancement of activity in both inhibition and disaggregation of Aβ_1–42_ amyloid fibrillation.

### 12III1 Phage As Capture Probe for AD-IgGs

In order to verify that 12III1 peptide could be a conformational mimotope of antigens exposed in amyloid aggregates in Alzheimer’s disease patients, we used the selected phage clone as putative capture probe for specific IgGs to discriminate between AD patients and healthy individuals.

These experiments were properly designed to evaluate the ability of this technology to provide alternative biomarker candidates for human disease, although at present it has some limitations to overcome. First, clinical criteria were used for diagnosis of AD, without the current possibility for us to analyze how serum IgG levels were related to biomarkers such as Aβ deposition or tau-mediated neuronal degeneration. Second, the sample size of each diagnostic group was relatively small, which we are expanding in studies in progress.

We carried out a preliminary study on 55 serum samples, 29 AD and 26 CTR. In a first step, 12III1 phage clone and pC89 wild-type vector (without peptide exposed) were used in indirect ELISA assay as antigen target for IgG sera of patients with Alzheimer’s disease and a control group without dementia signs. ELISA assays were optimized using several phage concentrations (1 × 10^12^, 1 × 10^11^, 1 × 10^10^) adsorbed onto 96-well microwell ELISA plates with reciprocal sera dilutions at 1:10, 1:50, 1:100 (data not shown). Optimal phage binding was found at 1:50 serum dilution in blocking buffer and at 10^11^ phage clone concentration (12III1 recombinant clone against wild-type vector pC89 without peptide exposed). The average of the data obtained with wild-type vector pC89 was used to calculate an arbitrary cutoff value (CO). Sera that showed an absorbance value less than 0.398 (CO = *N* × 3, where *N* is the average value of pC89) were considered as not responsive for 12III1.

**Figure 5:**
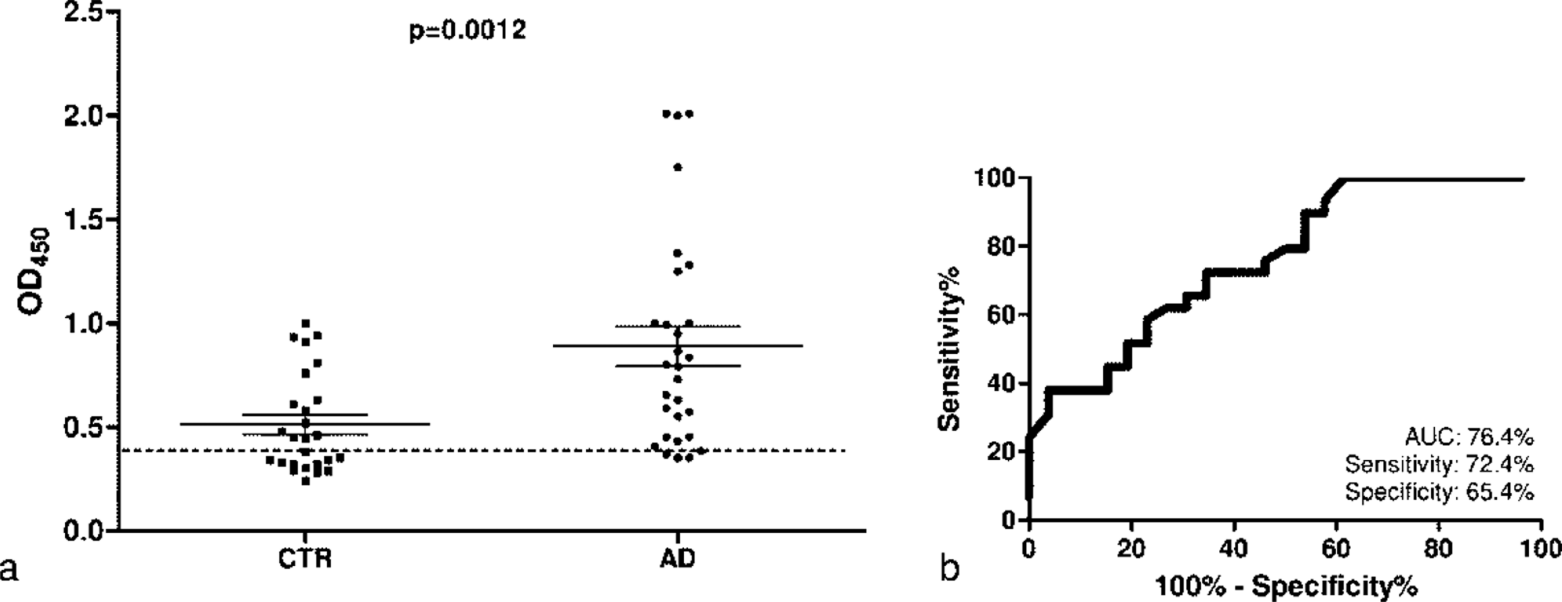
(a) Antibody binding to 12III1 phage clone in serum samples from patients with Alzheimer’s disease (AD) (*n* = 29) and healthy individuals (CTR) (*n* = 26). The dashed line represents the cutoff for positive binding (*N* × 3, where *N* is the average value of pC89). Data are reported as absorbance at 450 nm. Each single dot represents the mean value from triplicates of two independent experiments. (b) ROC curve analysis of the diagnostic performance of 12III1 phage clone. AUC, area under the curve. Sensitivity and specificity reported are those at the cutoff value corresponding to the point with the lowest distance to the upper-left corner of the ROC curve and giving the highest Youden’s Index: 95% confidence interval 0.64–0.89; *P* value 0.0008.

Obtained data are displayed in Figure 5. They showed IgG levels in AD patients significantly higher than in healthy controls (mean AD = 0.89, CTR = 0.51, with *p*-value = 0.0012, *R*^2^ = 0.18), providing a significant level of discrimination between diseased and nondemented subjects.

In particular, 25/29 AD sera (86.21%) and 14/26 CTR sera (53.85%) have IgGs recognized by 12III1 phage clone, whereas in 4 AD patients (13.79%) and in 12 control subjects (46.15%), IgG levels were below the pC89 cut off value.

Four of the control subjects, without apparent clinical AD signs, displayed a relatively high level of antibodies (similar to that found in AD patients). This may represent a possible false positive result, we suppose probably due to the cross-reaction of non-AD associated antibodies with this mimotope.

On the other hand, four AD patients were negative. As discussed above, this could be due to an incorrect diagnosis of Alzheimer’s disease. In fact, more recent clinical studies have demonstrated biomarker evidence of “suspected non-Alzheimer disease pathophysiology” (SNAP) causing amnestic type cognitive impairment with substantial hippocampal atrophy but lacking detectable β-amyloid amyloidosis.^4^ Indeed, other proteinopathy has been associated with substantial cognitive impairment that mimics AD clinical syndrome (i.e., limbic-predominant age-related TDP-43 encephalopathy, LATE).^5^

**Figure 6:**
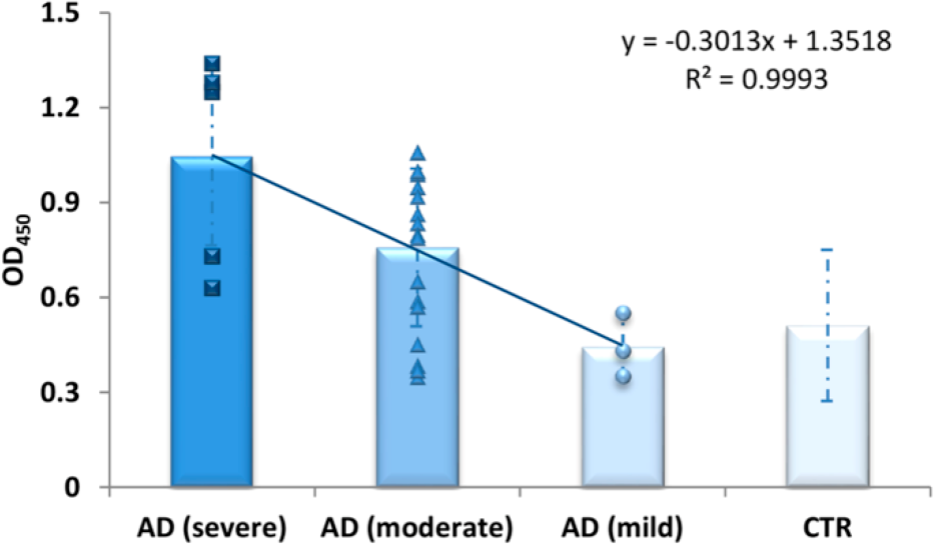
Antibody binding to 12III1 phage clone in serum samples from healthy individuals (CTR) (*n* = 26) and patients with Alzheimer’s disease (AD) (*n* = 29), reported as average values of absorbance at 450 nm within three disease stages according MMSE evaluation. Mild dementia: score of 20–24. Moderate dementia: score 12–20. Severe dementia: score less than 10. Error bars indicate standard deviations. The plotted trend line, the regression equation, and the *R*^2^ value are shown.

Since a small group of AD patients had IgG titers overlapping with the control subjects, we evaluated the possibility of finding a correlation with the disease stage evaluated on the basis of Mini Mental State Examination (MMSE) values (Figure 6).

Although based only on MMSE values, it emerges that IgG levels from the stratification of AD serum IgGs recognized by 12III1 are low in subjects with mild dementia, while they significantly rise in 88% of patients with moderate AD, and they are elevated in all sera with severe AD, suggesting this phage clone could be used as a probe for different Aβ polymorphisms typical of disease progression.

It has been reported that serum levels of Aβ autoantibodies are lower in AD patients than in healthy subjects,^14−16^ while others reported either higher values^13^ or no difference.^17,18^ Our results indicate that IgG levels detected by 12III1 phage clone are significantly higher as the disease progresses, suggesting that these IgG antibodies recognize other epitopes than Aβ-autoantibodies detected by several authors.^25,65^ On the other hand, a similar approach for discovery of IgG serum biomarkers not requiring prior knowledge of native antigens was used by Reddy et al.,^66^ and similar results were obtained using the selected synthetic peptoids to discriminate AD patients.

Further studies are in progress to expand the serological platforms in order to validate the preliminary results obtained.

## Conclusions

AD is today the main cause of dementia in the population, and it is associated with misfolding and aggregation states of β-amyloid. The polymorphic aggregation states of this peptide are correlated with the onset and progression of the disease. Diagnostic guidelines for AD include preclinical, MCI, and dementia evaluations as diagnostic stages. Currently, the preliminary diagnosis of AD is made by a combination of laboratory and clinical criteria, which include neuropsychiatric tests, behavioral history assessments, and neuroimaging techniques.^1−3^ The main biological markers of AD are the detection of amyloid and pathologic tau proteins. However, since changes in the brain can precede the appearance of the disease even of a considerable amount of time, new biomarkers are searched with ever increasing propensity as indicators of the beginning and progression of the pathological process. Currently, there are no predictive diagnostic systems capable of monitoring the disease. This will open the opportunity to identify new clinically relevant markers for detecting the state and stage of the disease.

In this study, we aimed to detect the presence of antibodies against “conformational epitopes” of the amyloid fibril aggregation states in AD patients that can be used as new state and stage markers for AD. The data obtained by using alternate biopanning with AD sera and mAb-YPF19 (monoclonal antibody for Caf1 protein of *Yersinia pestis*, chosen as like Aβ-amyloid structure protein), in the double binding phage display selection, showed that it was possible to identify alternative conformational mimotopes of Aβ-amyloid misfold typical of disease state and stage, as reported for 12III1 phage clone. Actually, this selected recombinant phage clone was able to interact *in vitro* with Aβ_1–42_ by both preventing aggregation and promoting disaggregation of preformed fibrils of the amyloid structure, significantly enhancing the already known M13 phage inhibitory activity. In addition, in a preliminary explorative study, this clone was able to detect the presence of specific antibodies in AD-sera. The proposed approach therefore proved to be a method for obtaining conformational mimotopes for proteins of interest, namely, for β-amyloid, that could be of putative diagnostic relevance in AD.

In summary, we have developed a technology based on double binding phage display selection for the discovery of phage displayed peptides capable of retaining IgG biomarkers from serum, that does not require preliminary knowledge of native antigens. Additionally, these phages exhibited a novel ability to increase the disaggregation of fibrils with future perspectives in therapy.

Further studies are in progress to validate the preliminary results obtained to suggest this technology to develop diagnostic tests for a variety of important human diseases.

## Methods

### Bioinformatics Analyses

The structural similarity search, to identify Aβ_1–42_-like conformational structures, consisted of the following steps:

Template selection. Template proteins were searched among proteins from different species with similar conformation or self-assembly process, using UniProtKB tool (https://www.uniprot.org/). For the structural similarity search, we placed main restrictions according to the known properties of amyloid oligomer conformations. First, the search was limited to only structures having a β strand, which is a generally accepted basic component of amyloid. Then, the search was limited to only proteins having no enzymatic or catalytic function or regulation activity, while having binary protein–protein interactions. To this aim, in advanced research, we set (i) in field UniProtKB AC Term “beta strand”, (ii) in field structure “3D structure available”, (iii) “NOT *Homo sapiens* (Human) [9606]”, (iv) “binary interaction” (v) “NOT function Function CC, enzyme classification, activity regulation, catalytic activity”. The use of UniProtKB tool has permitted identification of 47 proteins (Table S1 in Supporting Information).

Screening proteins. From selected proteins, excluding those uncharacterized or without identified 3D structure and those of infectious agents involved in common human diseases or commensal bacteria of human microbiome, F1 capsule antigen (UNIPROT ID P26948) was considered for the following steps in 3D alignment recognition to verify the match with Aβ structures.

Alignment homology. First of all, amino acid sequences of Aβ_1–42_ and F1 antigen were aligned using Clustal X2.1^67^ (Gonnet 250 Protein Weight Matrix, Gap opening 10, Gap extend 0.1), and alignment was analyzed using the GeneDoc (https://genedoc.software.informer.com/2.7/) tool to identify similar regions based on physicochemical properties of side chain amino acids.

Then, alignment of 3D protein structure was performed to verify the conformational match of F1 protein with Aβ structures. The PDB IDs associated with the 3D structure of the protein of interest were obtained from UniprotKB, through the ENTRY code P26948, associated with F1 capsule antigen. More PDB IDs are associated with F1 capsule antigen protein due to different 3D isolation and structure deposition of the protein (Table 3).

**Table 3:** PDB IDs Associated with F1 Capsule Antigen Protein

entry	protein name	organism	PDB IDs
P26948	F1 capsule antigen	*Yersinia pestis*	1p5u, 1p5v, 1z9s, 3dos, 3dpb, 3dsn, 4ayf, 4az8, 4b0m

PDB IDs associated with preassembly complexes (1p5v, 4ayf, 4az8, 4b0m) were excluded because Caf1 could not be in its mature conformational structure. The other PDB IDs were included in .txt file format. EMBL-EBI tool was used in PDBeFold section (http://www.ebi.ac.uk/msd-srv/ssm/), setting SSM submission form as follows: (i) query, PDB code 1z0q or 2nao for Aβ peptide (1–42) and amyloid fibril, respectively; (ii) target, list of PDB codes, upload PDB IDs of selected proteins in .txt file format; (iii) lowest acceptable match (%) for query, 100%, and lowest acceptable match (%) for target, from 0%; (iv) deselection if “match individual chain” to obtain the alignment with all the structure.

The 3D alignment with Aβ in fibril form (PDB ID 2nao) was carried out using Sequence & Structure Alignment tool available at https://www.rcsb.org/pages/analyze_features#Sequence, with algorithm JFATCAT rigid.

See section 1 of Supporting Information for further details.

### Human Samples

We retrospectively selected 29 sera (15 men and 14 women, mean age 70.7) of patients affected by AD diagnosed according to the criteria of the National Institute of Neurological and Communicative Disorders and Stroke and the Alzheimer’s Disease and Related Disorders Association,^68^ attending the Neurologic Unit of the University Hospital “Policlinico Vittorio Emanuele” in Catania. All AD patients underwent clinical and instrumental evaluations as normal diagnostic workup at the time of their admission. Presence and severity of cognitive impairment was assessed through the Mini Mental State Examination (MMSE).^69^ During the admission, a serum sample was collected and stored at −80 °C. At the time of admission, patients signed an informed consent allowing further use of the samples for diagnostic or research purposes.

Twenty-six healthy controls (12 men and 14 women, mean age 65.3) were recruited from subjects who accompanied patients for hospital check-ups. Healthy controls underwent a standard neurological examination performed by experienced neurologists in order to exclude the presence of neurological disorders and were enrolled in the study only after signing an informed consent. A serum sample was collected at the time of the enrolment and stored at −80 °C. The study was approved by the Ethic committee of the Policlinico Vittorio Emanuele of Catania.

### Double Binding Phage Display Selection

Phage M13 libraries were used, kindly donated by Prof. Franco Felici, expressing random peptides exposed on the pVIII protein, based on the phagemid vector pC89^70^ in which random oligonucleotide sequences were inserted in the region 5′ of the VIII gene present in the vector, under the control of the LacZ promoter. The digestion with the restriction enzymes *Eco*RI and *Bam*HI linearized the vector and allowed the insertion of the oligonucleotides with random sequences, which flanked by the same restriction sites allow the recircularization of the vector through ligase reaction. For the selection, two types of peptide libraries, expressing 12 amino acids in the pVIII amino terminal region, were used: pVIII-12aa and pVIII-12aa-Cys, wherein the latter peptides have a cysteine–cysteine constriction expressed in the peptide, so as to stabilize the structure thereof. The amplitude of each library comprises between 10 and 100 million independent clones.

As a source of antibodies directed against possible conformational epitopes of human polymorphic β-amyloid 1–42 (Aβ_1–42_), it was decided to use a pool of 5 human sera from patients with AD (IgG-AD) (mean age of 77.4 years, mean value of MMSE = 15.2). On the other hand, the monoclonal antibody YPF19 (AbDSerotec A Bio-Rad Company, IgG1-9820-5007) anti-*Yersinia pestis* F1 (reacting to *Y. pestis* F1 capsular antigen, Uniprot code P26948) was used to screen for putative conformational mimotopes homologous to Caf1 of *Yersinia pestis*, which results are of interest in the conformational structure similarity with Aβ_1–42_ as evoked by bioinformatics analysis.

It is known that filamentous bacteriophage M13 binds and disrupts a variety of misfolded protein assemblies, including Aβ, α-synuclein, and tau,^63^ so before using the M13 libraries, we verified reactivity of the wild-type vector pC89 (containing no insert) against antibodies used as bait in double binding phage display selection. It showed no reactivity against mAb YPF19 (mean 0.045, as the negative control in ELISA assay at every phage titer tested) and mild reactivity against AD-sera pool (mean 0.1326 in ELISA assay at 10^11^ phage/well) that was used to calculate the cutoff value in serum platforms of ELISA assays with recombinant phage clones obtained by selection.

For the immobilization of the antibodies, Dynabeads Protein G (Thermo Fischer scientific) supermagnetic beads of 2.8 μm were used. Buffers and reagents were purchased from Sigma-Aldrich.

Dynabeads Protein G (50 μL) were washed 3 times with citrate-phosphate buffer under stirring for 10 min, separated with a magnetic device for 1–2 min, and then incubated with sera pool containing IgG-AD diluted 1:10 or 5 μg of mAbYPF19 for 60 min at room temperature (RT) under mild stirring. Dynabeads functionalized with IgG-AD (DYN–IgG-AD) or mAbYPF19 (DYN–mAbYPF19) were separated with a magnetic device for 2 min, washed 4 times with Conjugation Buffer (20 mM sodium phosphate, 0.15 M NaCl, pH 7–9), separated with a magnetic device for 2 min and resuspended in 250 μL of 5 mM BS3 (bis(sulfosuccinimidyl)suberate) at RT for 30 min. Cross-linking reaction was blocked by adding 12.5 μL of Quenching Buffer, from 25 mM to 60 mM Tris, incubated at room temperature for 15 min with inclination and rotation. DYN–IgG-AD and DYN–mAbYPF19 were washed 3 times with 200 μL of PBST and finally resuspended in 1 mL of storage solution (PBS + 1% BSA + 0.01% Tween 20 pH 7.4).

Before using for phage display selection, beads were blocked for 1 h at room temperature with PBS (pH 7.4)–5% notfat milk–0.05% Tween 20.

#### Library Pretreatment

Each library (100 μL, 1 × 10^12^ viral particles) was added to 50 μL of Dynabeads Protein G and resuspended in 190 μL of TBS-Tween 0.1%. After 30 min incubation, the beads were separated with a magnetic device for 1–2 min; the supernatant was recovered and used to carry out again the two preceding steps twice before the use of the library for the selection.

#### Isolating Phages of Interest, Double Binding Procedure

**Figure 7:**
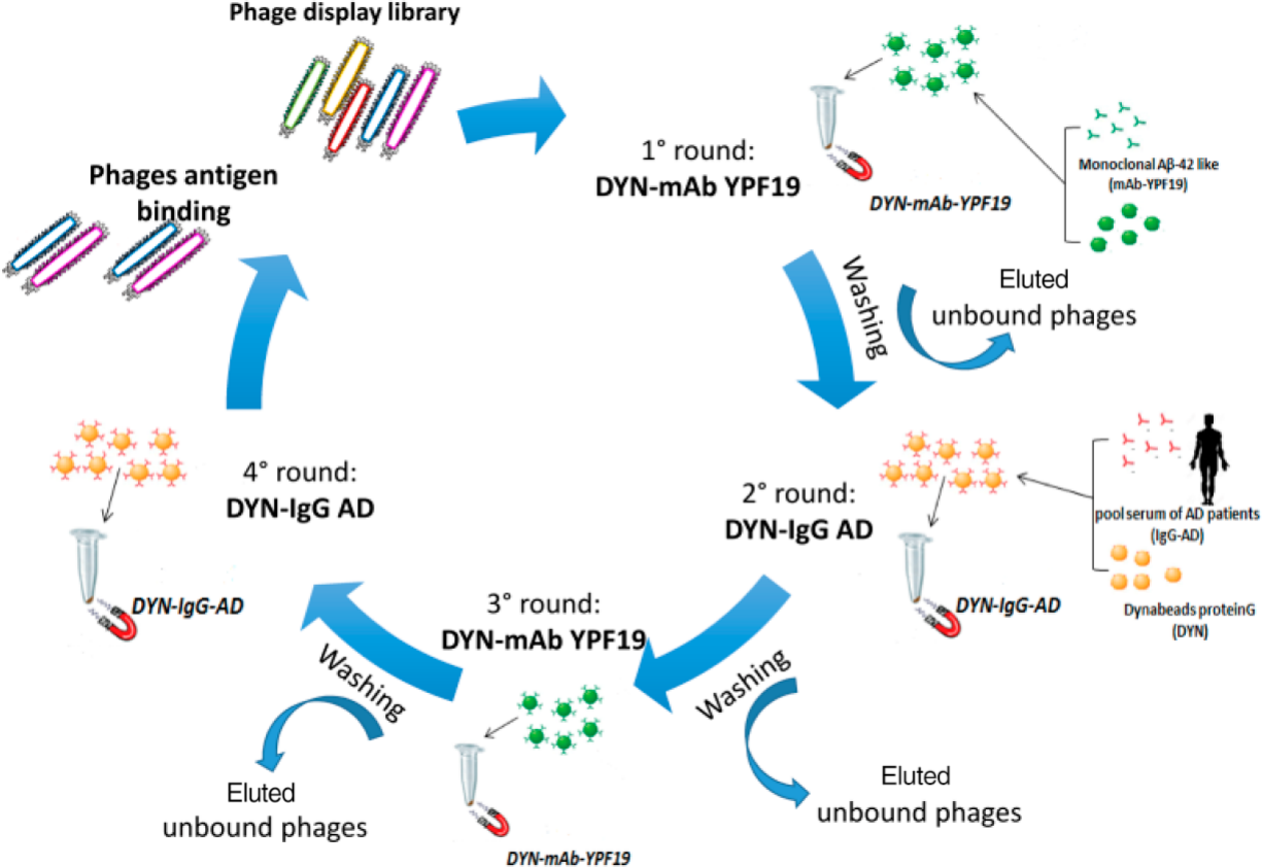
Scheme of double binding phage display selection.

In order to isolate phage expressing common sequences recognized both by the human sera pool IgG-AD and mAbYPF19, a selection procedure referred to as double binding was developed, as shown in Figure 7.

In brief, four selection cycles were carried out for each library, referred to as biopanning cycles. This cross-selection procedure allows, in the first round, to select the phage population capable of binding the monoclonal antibody YFP19 immobilized on the beads. The eluted phages in this step are used in the second selection round for screening against IgG-AD. In this case, only phage clones that cross react also with mAbYPF19 will have the possibility of binding the target, so as to limit the number of reactive phage to those common to the two antibody classes, IgG-AD and mAbYPF19. A third biopanning cycle against the mAbYPF19 again is then carried out with the eluted phages from the second selection round, and finally a fourth cycle against IgG-AD is carried out, in order to select phage pools having more affinity for the latter.

Briefly, in the first round of selection, 500 μL of DYN–mAb YPF19 were incubated with 100 μL of each of the four phage libraries with a titer of 10^12^ for 3–4 h at room temperature under mild stirring. The beads were washed 3 times in PBS–0.05% Tween 20 and separated with a magnetic device for 1–2 min to eliminate supernatants containing phage that did not bind the target present on the functionalized beads. Selected phage clones were eluted from antibodies with 500 μL of eluting buffer, 0.2 M of glycine-HCl (pH 2.2) + 0.1% BSA, neutralized immediately with 1 M Tris–HCl, pH 9.6. The enriched phage pools were amplified by infecting TG1 *E. coli*, purified twice by PEG precipitation, titrated, and used as the input for further panning. Biopanning affinity selection was repeated in the second round against DYN–IgG-AD, then in the third round against DYN–mAb YPF19 again, and finally a fourth selection round was carried out as the second one.

### Immunoscreening and ELISA Assays Using AD Sera

The phage clones coming from the third and fourth rounds were used in a double immunoscreening assay, which allows identification in a phage population of positive clones against the antibodies of interest.

Eluted phages (200 μL) were used to infect 800 μL of exponentially growing TG1 *E. coli* cells by incubation for 35 min at 37 °C. After infection, the bacteria were spread on a large LB agar plate containing ampicillin (50 mg/L) and 1% glucose. After overnight incubation at 37 °C, the colonies were recovered from the agar plate with a glass spreader, and 5–10 mL of LB broth containing ampicillin was added until a homogeneous suspension was obtained. Ten microliters of bacterial suspension was inoculated in 2 mL of LB with ampicillin to yield an OD_600_ value between 0.05 and 0.1. After growth up to an OD_600_ of 0.4 at 37 °C, 500 μL of culture was infected with 0.5 μL of M13KO7 helper phage (∼10^12^ TU/mL, Agilent Technologies) for 35 min at 37 °C. The infected bacteria were diluted, plated on ampicillin–kanamycin–IPTG-containing LB plates, and incubated overnight at 37 °C in order to obtain single colonies. For the dilution plates in which there is a number of colonies between 50 and 100, the protocol is carried out. Bacterial colonies, each containing a single phage clone population, were transferred onto a nitrocellulose (NC) membrane (Trans-Blot Transfer Medium 0.45 mm Bio-RAD) for 1 h at room temperature. Two filters in a sequence were prepared for each test. The NC membranes were blocked at room temperature for 1 h and 30 min in blocking solution (5% nonfat milk and 0.05% Tween 20 in PBS) under pivoting stirring and then washed by immersion in ultrapure sterile H_2_O. To check the reactivity against the AD sera pool used in phage display selection, the membranes were incubated with AD sera pool in PBS + 1% milk + 0.1% Tween 20 for 2 h at room temperature under pivoting stirring. After being washed 3 times in PBS–0.05% Tween 20, the membranes were incubated with HRP-conjugated anti-human IgG (IgG Fc AP113P) diluted 1:15000 in PBS + milk 1% + Tween 20 0.01%. After the membranes were washed as described above, positive spots on the immunoblots were detected using the Stable DAB chromogen system (Life Technologies, Monza, Italy). A second replicate lift was usually obtained and worked up in like manner testing immunoreactivity of the phage clones also to mAbYPF19: the same procedure was carried out coating the membranes with 5 μg/mL mAbYPF19 in PBS–1% milk–0.1% Tween 20 and developing with HRP-conjugated anti-mouse (Abcam ab97023) diluted 1:50000 in PBS + milk 1% + Tween 20 0.01%. Bacterial colonies, each producing a phage clone population, showing the highest signals in both membranes were individually amplified.

In order to confirm binding specificity for the monoclonal antibody YPF19 and the IgG-AD of the AD sera pool used for the selection, recombinant phage clones individually propagated from positive immunoscreening were tested for reactivity with the selecting antibodies by using an indirect ELISA assay. Phage preparations at a concentration of 10^12^ TU/mL were added in duplicate, 100 μL/well, into a 96-well microtiter plate (Multisorp, Nunc, Roskilde, Denmark). Plates were left overnight at 4 °C, blocked for 2 h at room temperature with blocking buffer (PBS–Tween 20 0.05%–6% nonfat milk), and washed in PBS–0.05% Tween 20. mAb YPF19 (100 μL) diluted 1:100 in dilution buffer (PBS–Tween 20 0.1%–1% nonfat milk) or 100 μL of AD sera pool diluted 1:50 in dilution buffer was added in duplicate into wells and incubated 1 h at 37 °C under stirring. The plates were washed 10 times as described above and exposed to HRP-conjugated anti-human IgG (IgG Fc AP113P) diluted 1:15000 in dilution buffer or anti-mouse diluted 1:50000 for 1 h at 37 °C under stirring. The plates were washed 5 times as described above and developed with TMB substrate, incubating in the dark for 30–45 min at room temperature, and stopped with 100 μL of 1 N HCl. Optical absorbance was recorded at 450 nm (Labsystem Multiskan Bichromatic).The TBS was used as a negative control (non-antigen-coated wells) for the evaluation of the background noise reaction caused by hydrophobic binding of immunoglobulin components in sample specimens to solid surfaces. Wild-type vector pC89 (containing no insert) was used as an internal control for evaluation of nonspecific binding through M13 coat protein interaction.

### Sequence Analysis of Selected Phage Clones

The selected clones that simultaneously show greater reactivity against the two antibody categories, IgG present in the serum of AD subjects and mAbYPF19 (specific monoclonal antibody for the identified protein F1 capsular antigen having a high degree of conformational similarity for fibrillar Αβ42), were sequenced, and the compatibility with the predicted 3D conformational homology regions was evaluated.

For the amplification and sequencing, sources of phage DNA were colonies of infected bacteria. The sequencing primers M13-40Rev (5′-GTTTTCCCAGTCACGAC-3′) and E24Fw (5′-GCTACCCTCGTTCCGATGCTGTC-3′) were obtained from Proligo, Sigma (Milan, Italy). A sample (1 μL) of the suspended colony was added to the PCR reaction tube, containing 49 μL of PCR mixture. The mix was then denatured in a thermal cycler for 10 min at 95 °C, and then 0.25 μL of my TAQ was added. Each sample was subjected to the following PCR cycles: 4 min at 94 °C; 25 cycles of 30 s at 94 °C, 30 s at 52 °C, and 30 s at 72 °C; 7 min at 72 °C]. PCR products were analyzed by agarose gel electrophoresis (1%, w/v, agarose, Sigma, Milan, Italy), and 35 μL of products was purified with the extraction Kit Nucleo Spin (Macherey-Nagel) and sequenced by DNA sequencing service of BMR Genomics (Padova, Italy) using the primer M13-40Rev.

The DNA sequences were translated into amino acids by using the “translate” program on the proteomics server of Swiss Institute of Bioinformatics Expert Protein Analysis System (ExPASy, http://www.expasy.ch/).

Sequence alignments were performed using the CLUSTAL X sequence alignment program^67^ (available at http://www.ebi.ac.uk/clustalw/). GeneDoc (https://genedoc.software.informer.com/2.7/) was used as a tool for visualizing, editing, and analyzing multiple sequence alignments of the peptides. Each cluster of similar peptides was then aligned as a group with the amino acid sequence of F1 antigen and Aβ_1–42_ to identify regions with amino acid composition similar to that of the peptides. Selected 12III1 peptide was also aligned individually with the sequence of F1 antigen and Aβ_1–42_ using Clustal X2.1^67^ (Gonnet 250 Protein Weight Matrix, Gap opening 10, Gap extend 0.1).

The epitope mapping algorithm PepSurf (http://pepitope.tau.ac.il/), an algorithm for the prediction of epitopes derived from combinatorial phage display libraries,^47^ was used to map 12III1 peptide onto the three-dimensional protein structure of F1 capsular antigen (chain C of the relevant PDB IDs) and Aβ_1–42_ (chain A of PDB ID 2nao), and the algorithm Mapitope was used to map the selected peptide flanked by the sequence of exposed pVIII protein. The GRANTHAM matrix was used since it is based on physicochemical properties, such as side chain composition, polarity, and molecular volume. The gap penalty accounting for unmatched peptide residues was set to the default value of −0.5. Library type is random amino acid, and the stop codon UAG is read as glutamine (in TG1 bacterial strain, this function is encoded by the gene supE, which suppresses the UAG stop codon inserting a tRNA-amino acid instead).

In order to obtain *in silico* structural predictions with atomic-level accuracy of the molecular interaction between 12III1 phage clone displayed peptide and Aβ_1–42_ fibril, the protein docking program ZDOCK was used. It uses a fast Fourier transform (FFT) to perform a 3D search of the spatial degrees of freedom between the two molecules,^71^ using electrostatic interactions or both electrostatic and solvation terms selecting for low-energy conformations.

ZDOCK server, available online at http://zdock.umassmed.edu/, permits one to upload the desired molecule in PDB format.

First, engineered 12III–pVIII was designed using MODELLER, version 9.20, software (available at https://salilab.org/modeller/), a computer program for comparative protein structure modeling. The user provides an alignment of a sequence (target) to be modeled with known related structures (template), and MODELLER automatically calculates a model containing all non-hydrogen atoms. The models were built as reported previously.^72^ Briefly, the PDB structure of pVIII protein (PDB ID 2mjz) was downloaded for M13 phage. In the context of the whole virus particle of 2mjz, pVIII Chain [1a] was chosen for modeling the engineered 12III–pVIII. The pVIII Chain [1a] was kept as a single pVIII protein in PDB format and used like a template. Then the amino acid sequence of engineered 12III-pVIII, 1-AEGEFRWPPHFEWHFDDGDPAKAAFNSLQASATEYIGYAWAMVVVIVGATIGIKLFKKFTSKAS-64, was written in FASTA format and converted to ALI format. The scripts of the MODELLER 9.20 software were modified with the file names of the engineered 12III–pVIII and with the PDB ID of the molecule template, and then a target–template alignment was constructed. Successively, MODELLER 9.20 software calculates a 3-D model of the target completely automatically, using its automodel class in PDB format. Several models were calculated for the same target; the best model was selected by picking the model with the lowest value of DOPE, which indicates the construction energy.

So in ZDOCK server, as “receptor” the PDB ID of the amyloid fibril form (2nao) was indicated and as the “ligand” the designed 3D molecule of the engineered 12III1–pVIII protein was uploaded. Then the server was run using the FFT-based protein docking. In brief, the ZDOCK algorithm searches exhaustively the entire rotational and translational space of the ligand with respect to the receptor (protein), which remains fixed at the origin. The rotational search is performed by explicitly rotating the ligand around each of its three Cartesian angles by a certain increment, 15°. For every rotation, the algorithm rapidly scans the translational space using FFT. The output given is the molecular docking prediction in PDB format. The models performed in PDB format were opened and processed using Molegro Molecular Viewer v1.2.0 (http://molexus.io/molegro-molecular-viewer/) highlighting the amino acids involved in ligand–receptor interaction by “Ligand Map” and the electrostatic contributions by “Energy Map”.

### 12III1 Phage Clone Ability to Discriminate Pool Sera of AD from Healthy Individuals (CTR)

An indirect ELISA was used as described above, against a pool of five human sera of AD subjects (mean age of 77.4 years, mean value of MMSE = 15.2) and a pool of five human sera of healthy age-matched individuals (CTR). Student *t* test was used to analyze difference between the two groups in PRISM software (GraphPad), and difference was considered significant if *p* < 0.05. Receiver operating characteristic (ROC) curves were drawn, and area under curve (AUC), confidence interval, and *P* value were calculated to evaluate the diagnostic performance, using GraphPad PRISM 5 software.

### Inhibition by 12III1 and pc89 of β-Amyloid Cytotoxicity

Unless otherwise stated, all compounds were obtained from Sigma-Aldrich. All other chemicals were of the highest commercial grade available. All stock solutions were prepared in nonpyrogenic saline (0.9% NaCl, Baxter, Milan, Italy). Aβ_1–42_ was purchased from Tocris Bioscience and was dissolved 1 mg/mL in sterile water. SH-SY5Y cells were purchase from ATCC (CRL-2266).

SH-SY5Y cells are a cloned subline of SK-N-SH cells originally established from a bone marrow biopsy of a neuroblastoma patient with sympathetic adrenergic ganglial origin.^73^ SH-SY5Y neuroblastoma cells can be differentiated into neuron-like cells displaying morphological and biochemical features of mature neurons. Furthermore, these cells display axonal expression of mature tau protein isoforms. In the light of this, we found the best overall neuronal differentiation was achieved using retinoic acid (RA) pretreated SH-SY5Y cells as previously described.^74^ Human neuroblastoma SH-SY5Y cells were obtained from American Type Culture Collection (ATCC CLR-2266) and were grown to monolayer in a culture medium containing Dulbecco’s minimal essential medium (DMEM) and Ham’s F12, modified with 2 mM l-glutamine and 1.0 mM sodium pyruvate and supplemented with fetal bovine serum (FBS) to 10% and streptomycin 50 mg/mL. SH-SY5Y cells were maintained at 37 °C and 5% CO_2_.

For cell viability tests, 3 × 10^4^ cells were plated in 96-well plates (Corning Cell Culture) in a volume of 150 μL and differentiated with RA (100 nM) for 24 h. In order to test the inhibition of Aβ aggregation, different titers (10^6^, 10^8^ and 10^10^ TU/ml) of 12III1 phage clone or pC89 wild-type phage were added simultaneously to Aβ_1–42_ peptide 1 μg/mL, and cell viability was assayed after 24h incubation. For evaluation of cytotoxicity inhibition by phages on preassembled Aβ, differentiated SH-SY5Y cells were stimulated with Aβ_1–42_ peptide, 1 μg/mL, for 3, 6, or 12 h and then, at each time, incubated with the different titers of 12III1/pC89 phage for additional 24 h.

Three sequential rounds of precipitation in 4% (w/v) PEG 8000, 500 mM NaCl supplemented with 2% (v/v) Triton X-100 were carried out to purify phage clones and remove LPS contamination, according Branston et al.^75^

Cell viability was evaluated using the mitochondria-dependent dye 3-(4,5-dimethylthiazol-2-yl)-2,5-diphenyltetrazolium bromide (MTT) colorimetric assay as previously described,^76^ expressed as % viability versus control cells grown in normal culture medium (Ctr). Cultures pretreated with increasing concentrations of the test compound were incubated at 37 °C with MTT (0.2 mg/mL) for 1 h. Medium was removed, and the cells were lysed with dimethyl sulfoxide (100 μL). The extent of reduction of MTT to formazan was quantified by measurement of optical density at 550 nm with a microplate reader.

Statistical analysis was performed by one way ANOVA to analyze difference among groups using PRISM software (GraphPad), and difference was considered significant if *p* < 0.05.

### ELISA Assay of 12III1 Phage Clone against Single AD and Healthy Subject Sera (CTR)

Twenty-nine patients with AD with a MMSE index between 22 and 6.8 and 26 healthy nondemented control individuals were recruited, and the samples were tested according to a phage ELISA assay as follows. Indirect ELISA test was standardized for 12III1 clone and pC89 vector, adsorbing on the bottom of the wells logarithmic scale dilutions of phage (1 × 10^12^, 1 × 10^11^, and 1 × 10^10^ phage final concentrations) and then using 1:10, 1:50, and 1:100 dilutions of sera. The procedure gave maximal phage specific binding for all sera used with phage concentration at 1 × 10^11^ TU/well and serum dilution at 1:50.

Phage preparations were added in duplicate, 100 μL/well, into a 96-well microtiter plate (Multisorp, Nunc, Roskilde, Denmark). Plates were left overnight at 4 °C, blocked for 2 h at room temperature with blocking buffer (PBS–Tween 20 0.05%–6% nonfat milk), and washed in PBS–0.05% Tween 20. AD or CTR serum (200 μL of 1:50 dilution) was added and incubated 2 h at 37 °C. The plates were washed 10 times as described above and exposed to HRP-conjugated anti-human IgG (IgG Fc AP113P) diluted 1:15000 in dilution buffer for 1 h at 37 °C. The plates were washed 5 times as described above and developed with TMB substrate, incubating in the dark for 30–45 min at room temperature, and stopped with 100 μL of 1 N HCl. Optical absorbance was recorded at 450 nm (Labsystem Multiskan Bichromatic). TBS was used as a negative control for the evaluation of the nonspecific binding background. The cutoff value (CO = 0.398) was calculated as CO = *N* × 3 where *N* is the average of the data obtained with wild-type vector pC89.

One way ANOVA was used to analyze difference among groups using PRISM software (GraphPad), and difference was considered significant if *p* < 0.05.

No unexpected or unusually high safety hazards were encountered.

## Supporting Information Available

Structural similarity search results, PDBeFold analysis result summary and images, RCSB Sequence & Structure Aβ_1–42_–F1 alignment analysis images, and bioinformatics analysis of 12III1 phage clone (PDF)

## Abbreviations

AD: Alzheimer’s disease
Caf1: F1 capsular antigen
mAb: monoclonal antibody
MCI: mild cognitive impairment
SNAP: suspected non-Alzheimer disease pathophysiology
LATE: limbic-predominant age-related TDP-43 encephalopathy
CSF: cerebrospinal fluid
Aβ: amyloid beta
Aβ_1–42_: amyloid beta isoform of 42 amino acids
mOC: OC-type monoclonal antibody
OC: rabbit polyclonal antiserum that binds to fibrillar Aβ and other amyloid-like protein structures
CTR: sera of healthy individuals
GAIM: general amyloid interaction motif
CO: cutoff value
MMSE: Mini-Mental State Examination
DYN-IgG-AD: Dynabeads functionalized with IgG-AD
DYN-mAbYPF19: Dynabeads functionalized with mAbYPF19
PBST: phosphate-buffered saline with Tween 20 detergent
IPTG: isopropyl β-d-1-thiogalactopyranoside
HRP: horseradish peroxidase
DAB: 3,3-diaminobenzidine, HRP substrate
TMB: 3,3′,5,5′-tetramethylbenzidine in buffer for ELISA development
TBS: Tris-buffered saline
FFT: fast Fourier transform
MTT: 3-(4,5-dimethylthiazol-2-yl)-2,5-diphenyltetrazolium bromide
Ctr: control cells grown in normal culture medium.
TU: transducing units
