# Supplementary material for: Innovative IgG Biomarkers Based on Phage Display Microbial Amyloid Mimotope for State and Stage Diagnosis in Alzheimer’s Disease
Source: ACS Chem Neurosci. 2020 Mar 16;11(7):1013–26. doi: 10.1021/acschemneuro.9b00549 (PMC7997372; doi:10.1021/acschemneuro.9b00549)
Supplement: Supplementary file 1 — cn9b00549_si_001.pdf [file cn9b00549_si_001.pdf]

Supporting Information

Innovative IgG Biomarkers Based on Phage Display Microbial Amyloid Mimotope for State and Stage Diagnosis in Alzheimer's Disease

Laura M. De Plano[a,§], Santina Carnazza[a,§], Domenico Franco[a], Maria Giovanna Rizzo[a], Sabrina Conoci[b,c], Salvatore Petralia[b], Alessandra Nicoletti[d], Mario Zappia[d], Michela Campolo[a], Emanuela Esposito[a], Salvatore Cuzzocrea[a] and Salvatore P.P. Guglielmino[a,*]

[a]Department of Chemical, Biological, Pharmaceutical and Environmental Sciences, University of Messina, Viale F. Stagnod'Alcontres 31, 98166 Messina, Italy
[b]STmicroelectronics, Stradale Primosole, 50, 95121 Catania, Italy
[c]Distretto Tecnologico Micro e Nano Sistemi Sicilia, Strada VII-Zona Industriale, 95121 Catania, Italy
[d]Neurology Clinic, Department "G.F. Ingrassia", Section of Neurosciences, University of Catania, Via Santa Sofia 78, 95123, Catania, Italy

*Corresponding Author: E-mail: sguglielm@unime.it; Phone +390906765198
ORCID 0000-0002-0401-6400

[§]Equal contribution

Table of Contents

Supplemental Data.......... 2

Table S1 Structural similarity search results by UniProtKB..........2

S1 PDBeFold analysis results. 4

S2 RCSB Sequence & Structure Aβ42-F1 Alignment analysis results..........7

S3 Bioinformatics analysis of 12III1 phage clone..........15

Supplemental Data

Table S1

Table S1. Structural similarity search results by UniProtKB

ENTRY	Protein names	Organism
**P49278**	Mite group 2 allergen Der p 2 (Allergen Der p II) (DPX) (allergen Der p 2)	*Dermatophagoides pteronyssinus* (European house dust mite)
**P38713**	Oxysterol-binding protein homolog 3	*Saccharomyces cerevisiae* (strain ATCC 204508 / S288c) (Baker's yeast)
**P43603**	LAS seventeen-binding protein 3 (LAS17-binding protein 3)	*Saccharomyces cerevisiae* (strain ATCC 204508 / S288c) (Baker's yeast)
**P0A7L8**	50S ribosomal protein L27 (Large ribosomal subunit protein bL27)	*Escherichia coli* (strain K12)
**Q9QYE9**	Pleckstrin homology domain-containing family B member 1 (PH domain-containing family B member 1) (Evectin-1) (PH domain-containing protein in retina 1) (PHRET1) (Pleckstrin homology domain retinal protein 1)	*Mus musculus* (Mouse)
**O04567**	Probable inactive receptor kinase At1g27190	*Arabidopsis thaliana* (Mouse-ear cress)
**P0A7N4**	50S ribosomal protein L32 (Large ribosomal subunit protein bL32)	*Escherichia coli* (strain K12)
**P0A7M2**	50S ribosomal protein L28 (Large ribosomal subunit protein bL28)	*Escherichia coli* (strain K12)
**Q9KWN0**	Actinohivin	*Actinomycete* sp. (strain K97-0003)
**P25638**	TPR repeat-containing protein associated with Hsp90	*Saccharomyces cerevisiae* (strain ATCC 204508 / S288c) (Baker's yeast)
**O14368**	Heat shock protein 16 (16 kDa heat shock protein)	*Schizosaccharomyces pombe* (strain 972 / ATCC 24843) (Fission yeast)
**Q12334**	Protein SCM3 (Suppressor of chromosome missegregation protein 3)	*Saccharomyces cerevisiae* (strain ATCC 204508 / S288c) (Baker's yeast)
**O13985**	Uncharacterized WD repeat-containing protein C26H5.03	*Schizosaccharomyces pombe* (strain 972 / ATCC 24843) (Fission yeast)
**P43672**	ABC transporter ATP-binding protein uup	*Escherichia coli* (strain K12)
**Q12335**	Protoplast secreted protein 2	*Saccharomyces cerevisiae* (strain ATCC 204508 / S288c) (Baker's yeast)
**P19243**	18.1 kDa class I heat shock protein (HSP 18.1)	*Pisum sativum* (Garden pea)
**P79085**	Major allergen Alt a 1 (allergen Alt a 1)	*Alternaria alternata* (*Alternaria rotfungus*) (*Torula alternata*)
**P9WGD5**	Single-stranded DNA-binding protein (SSB)	*Mycobacterium tuberculosis* (strain ATCC 25618 / H37Rv)
**P0AGK4**	RNA-binding protein YhbY	*Escherichia coli* (strain K12)
**P9WLZ7**	Uncharacterized protein Rv1364c	*Mycobacterium tuberculosis* (strain ATCC 25618 / H37Rv)
**P77667**	Protein SufA	*Escherichia coli* (strain K12)
**P04122**	Lactin beta-1 and beta-2 chains	*Lathyrus ochrus* (Yellow-flowered pea) (*Pisum ochrus*)
**P0A1J5**	Flagellar hook-associated protein 1 (HAP1)	*Salmonella typhimurium* (strain LT2 / SGSC1412 / ATCC 700720)
**P46973**	Protein HIT1	*Saccharomyces cerevisiae* (strain ATCC 204508 / S288c) (Baker's yeast)
**P0A917**	Outer membrane protein X	*Escherichia coli* (strain K12)
**P18481**	Trypsin-resistant surface T6 protein (T6 antigen)	*Streptococcus pyogenes* serotype M6 (strain ATCC BAA-946 / MGAS10394)
**O36030**	Uncharacterized WD repeat-containing protein C4F10.18	*Schizosaccharomyces pombe* (strain 972 / ATCC 24843) (Fission yeast)
**P9WFN1**	UPF0098 protein Rv2140c	*Mycobacterium tuberculosis* (strain ATCC 25618 / H37Rv)

**Q9WYC4**	Uncharacterized ABC transporter ATP-binding protein TM_0288	*Thermotoga maritima* (strain ATCC 43589 / MSB8 / DSM 3109 / JCM 10099)
**P27862**	IMPACT family member YigZ	*Escherichia coli* (strain K12)
**P0A1J1**	Flagellar hook protein FlgE	*Salmonella typhimurium* (strain LT2 / SGSC1412 / ATCC 700720)
**O59188**	Uncharacterized HTH-type transcriptional regulator PH1519	*Pyrococcus horikoshii* (strain ATCC 700860 / DSM 12428 / JCM 9974 / NBRC 100139 / OT-3)
**P26948**	F1 capsule antigen	*Yersinia pestis*
**Q9FX77**	B3 domain-containing protein At1g16640	*Arabidopsis thaliana* (Mouse-ear cress)
**P0AAN1**	Hydrogenase-2 operon protein HybE	*Escherichia coli* (strain K12)
**Q57399**	Uncharacterized ABC transporter ATP-binding protein HI_1470	*Haemophilus influenzae* (strain ATCC 51907 / DSM 11121 / KW20 / Rd)
**Q58105**	Uncharacterized NOP5 family protein MJ0694	*Methanocaldococcus jannaschii* (strain ATCC 43067 / DSM 2661 / JAL-1 / JCM 10045 / NBRC 100440) (*Methanococcus jannaschii*)
**P76049**	Uncharacterized protein YcjY	*Escherichia coli* (strain K12)
**P12307**	Mannose/glucose-specific lectine alpha 2 chain	*Lathyrus ochrus* (Yellow-flowered pea) (Pisum ochrus)
**Q60381**	Uncharacterized nitrogen regulatory PII-like protein MJ0059	*Methanocaldococcus jannaschii* (strain ATCC 43067 / DSM 2661 / JAL-1 / JCM 10045 / NBRC 100440) (*Methanococcus jannaschii*)
**P9WFK1**	UPF0336 protein Rv0635	*Mycobacterium tuberculosis* (strain ATCC 25618 / H37Rv)
**P0A8J4**	UPF0250 protein YbeD	*Escherichia coli* (strain K12)
**P07166**	Protein virC2	*Agrobacterium fabrum* (strain C58 / ATCC 33970) (*Agrobacterium tumefaciens* (strain C58))
**P04489**	Probable early E4 11 kDa protein	*Human adenovirus* C serotype 5 (HAdV-5) (*Human adenovirus* 5)
**P39332**	RutC family protein YjgH	*Escherichia coli* (strain K12)
**P0AD21**	Uncharacterized protein YejG	*Escherichia coli* (strain K12)
**O31697**	Uncharacterized protein YkzF	*Bacillus subtilis* (strain 168)

S1 PDBeFold analysis results

Results summary of PDBeFold alignments between Aβ Peptide (1-42) (PDB ID 1z0q) or amyloid fibril (PDB ID 2nao) and all the structures associated to F1 capsule antigen protein are shown below.

RESULT SUMMARY

##	Q-score	P-score	Z-score	RMSD	Nalgn	Nsse	Ngaps	Seq-%	Nmd	Nres-Q	Nsse-Q	Nres-T	Nsse-T	Query	Target
1	0.007328	1.956	4.104	2.389	13	1	1	0	1	42	2	336	20	PDB 1z0q	PDB 1p5u
2	0.006861	1.948	4.096	1.469	13	1	3	0	1	42	2	473	20	PDB 1z0q	PDB 3dpb
3	0.006658	1.906	4.053	1.581	13	1	3	0	1	42	2	473	20	PDB 1z0q	PDB 1z9s
4	0.005144	0.3422	2.083	2.601	19	1	2	0.1579	0	42	2	954	47	PDB 1z0q	PDB 3dos
5	0.004677	1.308	3.531	2.916	19	1	3	0	2	42	2	945	47	PDB 1z0q	PDB 3dsn

RESULT SUMMARY

##	Q-score	P-score	Z-score	RMSD	Nalgn	Nsse	Ngaps	Seq-%	Nmd	Nres-Q	Nsse-Q	Nres-T	Nsse-T	Query	Target
1	0.02697	-0	0.01646	5.775	123	2	24	0.03252	10	252	12	473	20	PDB 2nao	PDB 1z9s
2	0.02125	-0	0.3513	4.943	97	2	21	0.07216	8	252	12	473	20	PDB 2nao	PDB 3dpb
3	0.01036	-0	0.04359	5.861	109	4	23	0.09174	11	252	12	945	47	PDB 2nao	PDB 3dsn
4	0.01009	-0	0.0282	5.373	101	4	26	0.06931	14	252	12	954	47	PDB 2nao	PDB 3dos
5	0.008651	-0	0.04478	6.429	64	3	10	0.0625	5	252	12	336	20	PDB 2nao	PDB 1p5u

The following imagines show 3D structure of beta amyloid fibril (PDB ID 2nao) with the F1 capsule antigen (5 different PDB ID number) with highlighted amino acid positions involved in the homology.

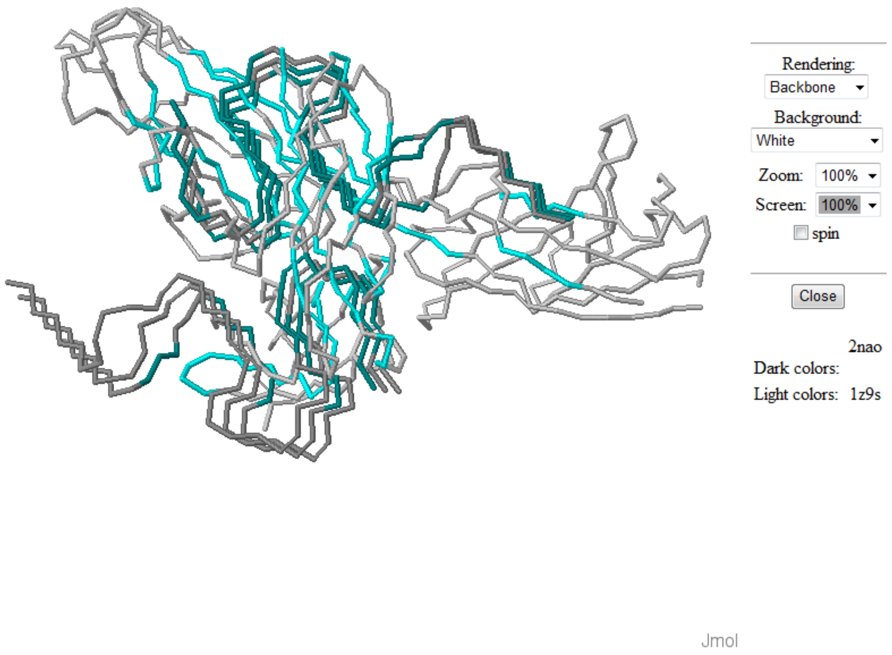

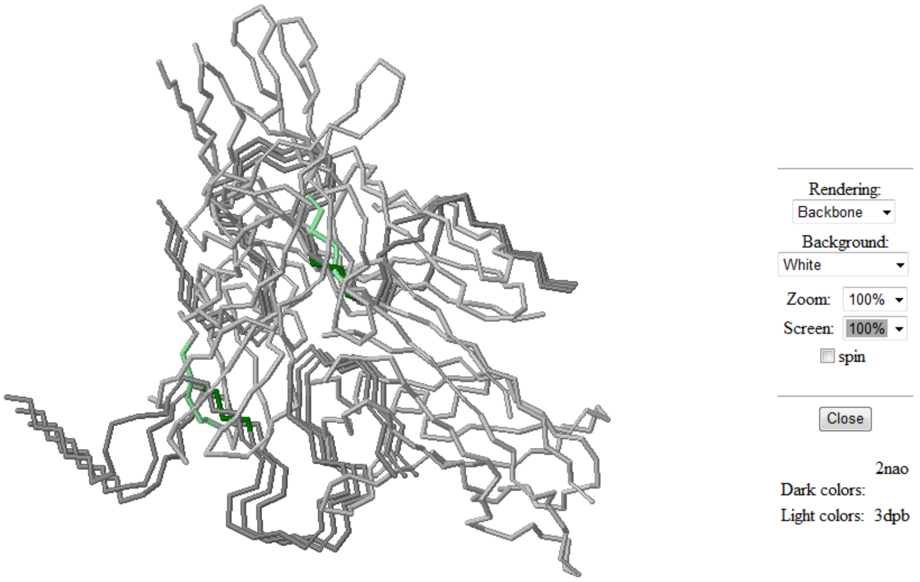
Rendering:
Backbone
Background:
White
Zoom: 100%
Screen: 100%
spin
Close
2nao
Dark colors:
Light colors: 3dpb

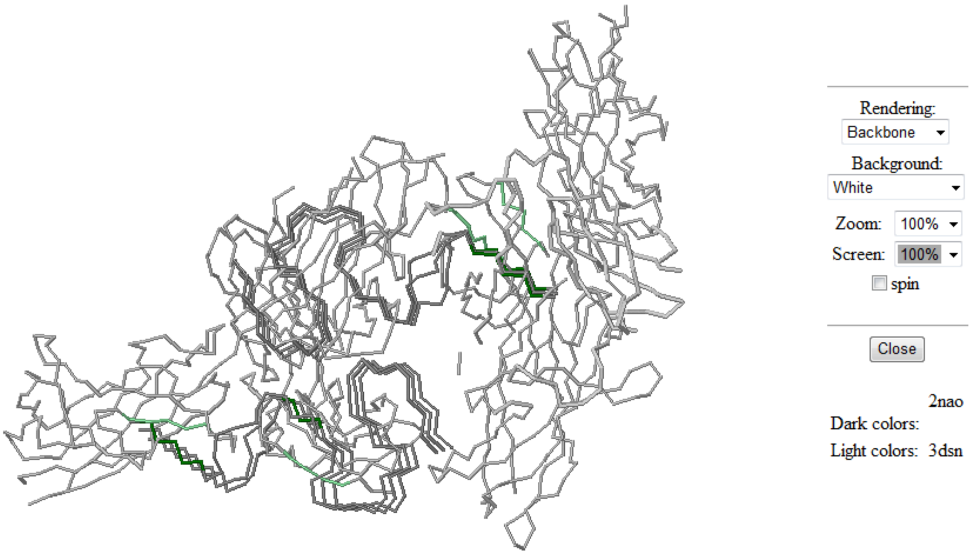
Rendering:
Backbone
Background:
White
Zoom: 100%
Screen: 100%
spin
Close
2nao
Dark colors:
Light colors: 3dsn

Jmol

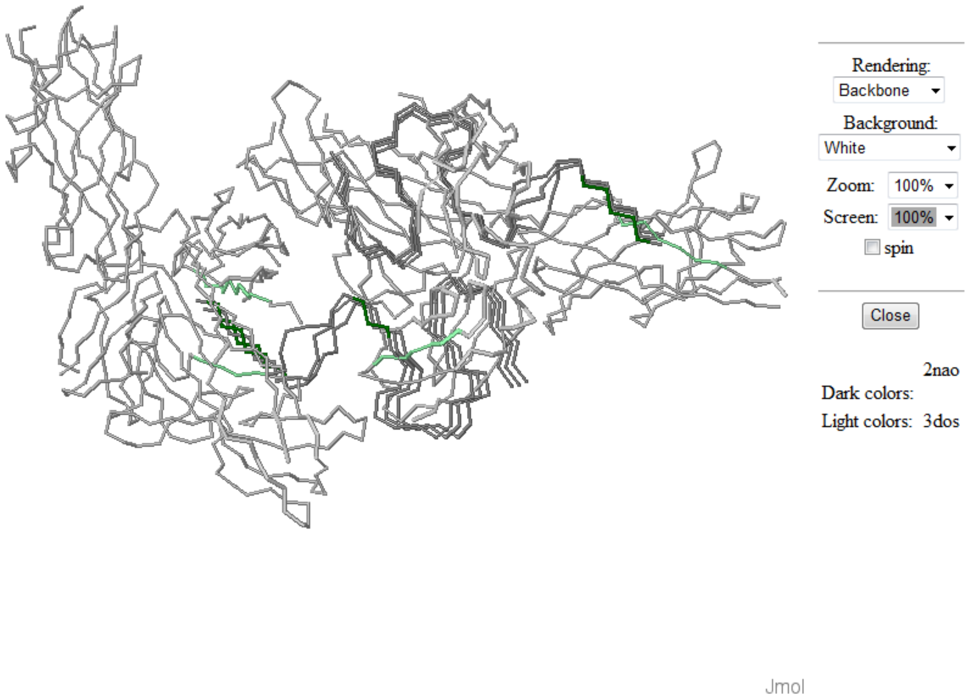
Rendering:
Backbone
Background:
White
Zoom: 100%
Screen: 100%
spin
Close
2nao
Dark colors:
Light colors: 3dos
Jmol

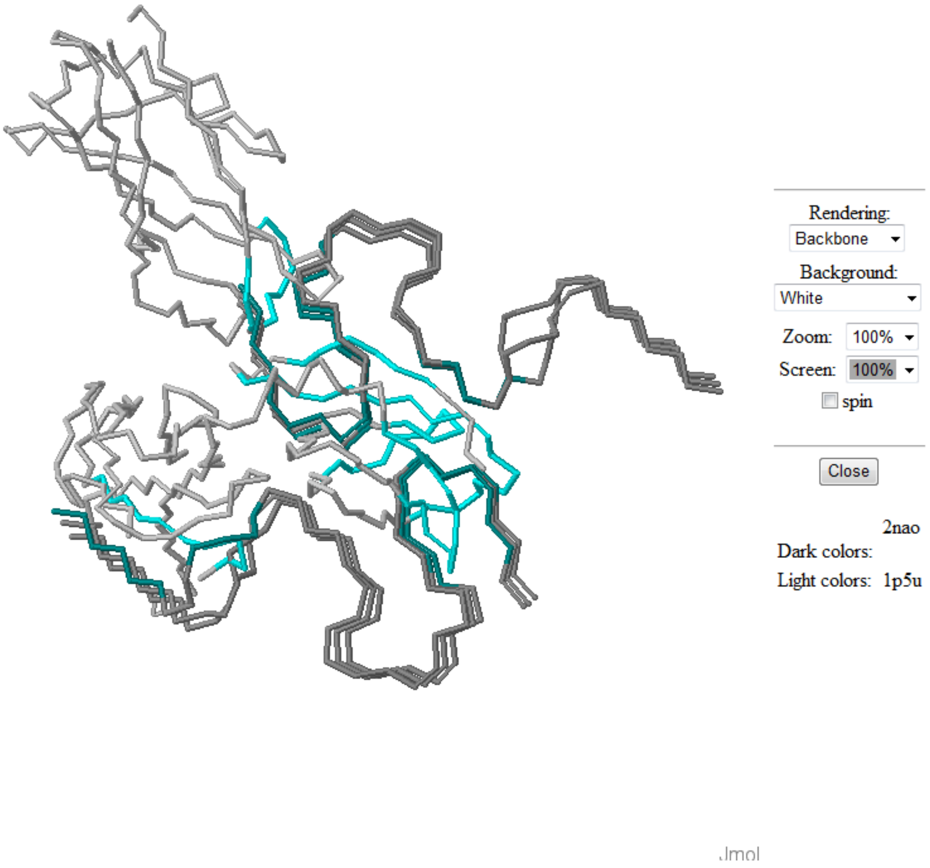
Rendering:
Backbone
Background:
White
Zoom: 100%
Screen: 100%
spin
Close
2nao
Dark colors:
Light colors: 1p5u
Jmol

S2 RCSB Sequence & Structure Aβ42-F1 Alignment analysis results

Alignment of Amyloid fibrils (PDB ID 2nao) with F1 capsule antigen using "Sequence and Structure Alignment" (https://www.rcsb.org/pages/analyze_features#Sequence)- algorithm JFATCAT rigid.

Structure Alignment Results

AlignmentDetails:Query: (orange/dark grey) **Subject:** (cyan/light grey)

		Beta-amyloid protein 42			F1 capsule antigen	
P-value:9.68e-01<br>Score:24.68<br>RMSD:3.58<br>%Id:0.0%	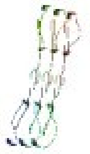	PDB ID:<br>Chain ID:<br>Length:<br>Similarity:	2NAO<br>A<br>42<br>33%	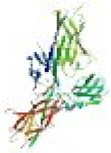	PDB ID:<br>Chain ID:<br>Length:<br>Similarity:	1Z9S<br>C<br>128<br>11%

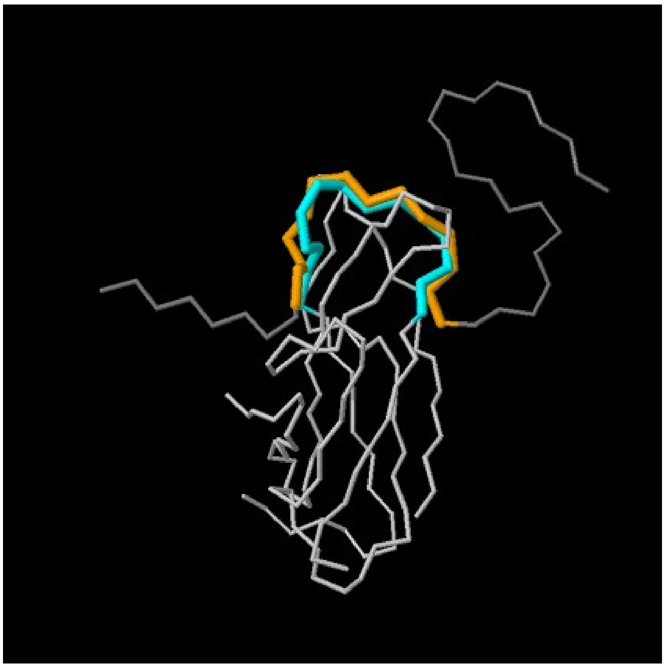

```
Align 2NAO.A.pdb Length1: 42 with 1Z9S.C.pdb Length2: 128
P-value: 9.68e-01
Equ: 14
RMSD: 3.58
Score: 24.68
Align-len: 14
Gaps: 0 (0.00%)
```

```
Identity: 0.00%
Similarity: 7.14%

9:A
||    .    |
GYEVHHQKLVFFAE
..............
IGSKGGKLAAGKYT
|   |    .
126:C



| ... Structurally equivalent and identical residues
: ... Structurally equivalent and similar residues
. ... Structurally equivalent, but not similar residues.

     To calculate the coordinates of chain 2 aligned on chain 1 apply the following tra
nsformation:

     X1 = ( 0.871949)*Xorig + (-0.484268)*Yorig + (-0.072045)*Zorig + (   28.995000)
     Y1 = ( 0.450925)*Xorig + ( 0.851654)*Yorig + (-0.267118)*Zorig + (  -81.550000)
Z1 = ( 0.190714)*Xorig + ( 0.200426)*Yorig + ( 0.960967)*Zorig + (  -78.219000)
```

Structure Alignment Results

AlignmentDetails:Query: (orange/dark grey) **Subject:** (cyan/light grey)

	Beta-amyloid protein 42			***F1 capsule antigen***		
P-value:9.67e-01<br>Score:24.73<br>RMSD:3.57<br>%Id:0.0%	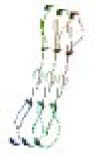	PDB ID:<br>Chain ID:<br>Length:<br>Similarity:	2NAO<br>A<br>42<br>33%	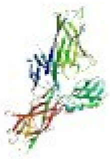	PDB ID:<br>Chain ID:<br>Length:<br>Similarity:	3DPB<br>C<br>128<br>11%

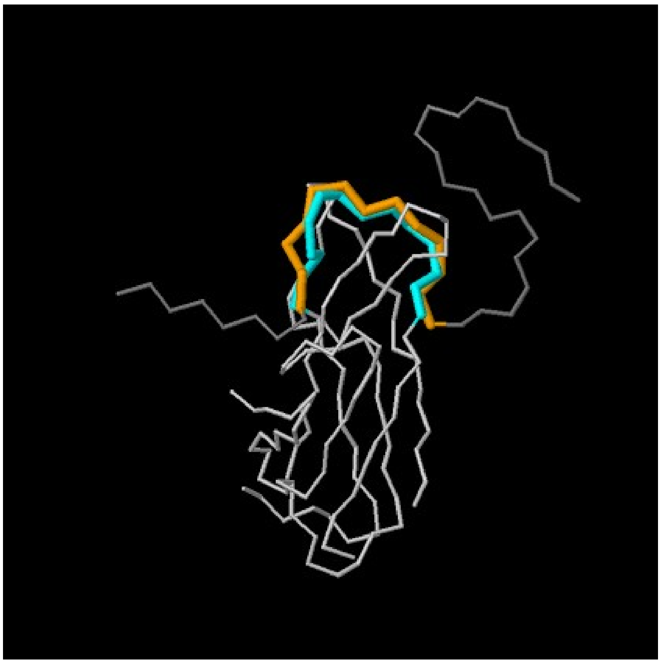

```
Align 2NAO.A.pdb Length1: 42 with 3DPB.C.pdb Length2: 128

P-value: 9.67e-01

Equ: 14

RMSD: 3.57

Score: 24.73

Align-len: 14

Gaps: 0 (0.00%)


Identity: 0.00%

Similarity: 7.14%


9:A

||    .    |

GYEVHHQKLVFFAE

..............

IGSKGGKLAAGKYT

|   |     .

126:C


| ... Structurally equivalent and identical residues

: ... Structurally equivalent and similar residues

. ... Structurally equivalent, but not similar residues.
```

```
     To calculate the coordinates of chain 2 aligned on chain 1 apply the following tra
nsformation:


     X1 = ( 0.875637)*Xorig + (-0.479433)*Yorig + (-0.058342)*Zorig + (  -15.339000)

     Y1 = ( 0.449539)*Xorig + ( 0.853220)*Yorig + (-0.264442)*Zorig + (   -4.946000)

Z1 = ( 0.176561)*Xorig + ( 0.205328)*Yorig + ( 0.962635)*Zorig + (  -59.838000)
```

Structure Alignment Results

AlignmentDetails:Query: (orange/dark grey) **Subject:** (cyan/light grey)

	Beta-amyloid protein 42			***F1 capsule antigen***		
P-value:9.54e-01<br>Score:23.15<br>RMSD:3.27<br>%Id:0.0%	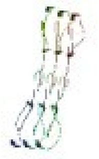	PDB ID:<br>Chain ID:<br>Length:<br>Similarity:	2NAO<br>A<br>42<br>43%	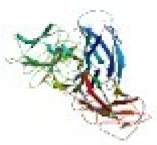	PDB ID:<br>Chain ID:<br>Length:<br>Similarity:	3DSN<br>C<br>132<br>14%

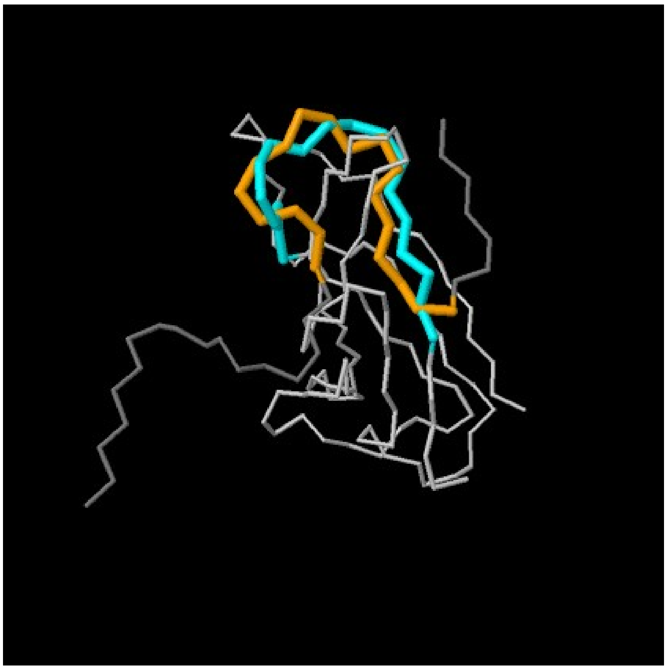

```
Align 2NAO.A.pdb Length1: 42 with 3DSN.C.pdb Length2: 132

P-value: 9.54e-01

Equ: 18

RMSD: 3.27

Score: 23.15

Align-len: 18

Gaps: 0 (0.00%)
```

```
Identity: 0.00%

Similarity: 16.67%


18:A         30:A

| |    .    |    .

VFFAEDVGSNKGAIIGLM

:.................

IGSKGGKLAAGKYTDAVT

|   |    .    |

126:C         140:C


| ... Structurally equivalent and identical residues

: ... Structurally equivalent and similar residues

. ... Structurally equivalent, but not similar residues.


     To calculate the coordinates of chain 2 aligned on chain 1 apply the following tra
nsformation:


     X1 = ( 0.949283)*Xorig + ( 0.254346)*Yorig + (-0.184854)*Zorig + (   -31.919000)

     Y1 = ( 0.055223)*Xorig + ( 0.443907)*Yorig + ( 0.894370)*Zorig + (   -25.467000)

     Z1 = ( 0.309537)*Xorig + (-0.859218)*Yorig + ( 0.407347)*Zorig + (   -49.574000)
```

Structure Alignment Results

AlignmentDetails:Query: (orange/dark grey) **Subject:** (cyan/light grey)

	Beta-amyloid protein 42			***F1 capsule antigen***		
P-value:9.60e-01<br>Score:23.19<br>RMSD:4.29<br>%Id:0.0%	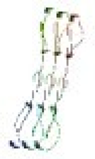	PDB ID:<br>Chain ID:<br>Length:<br>Similarity:	2NAO<br>A<br>42<br>52%	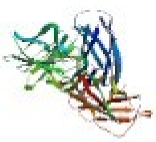	PDB ID:<br>Chain ID:<br>Length:<br>Similarity:	3DOS<br>C<br>135<br>16%

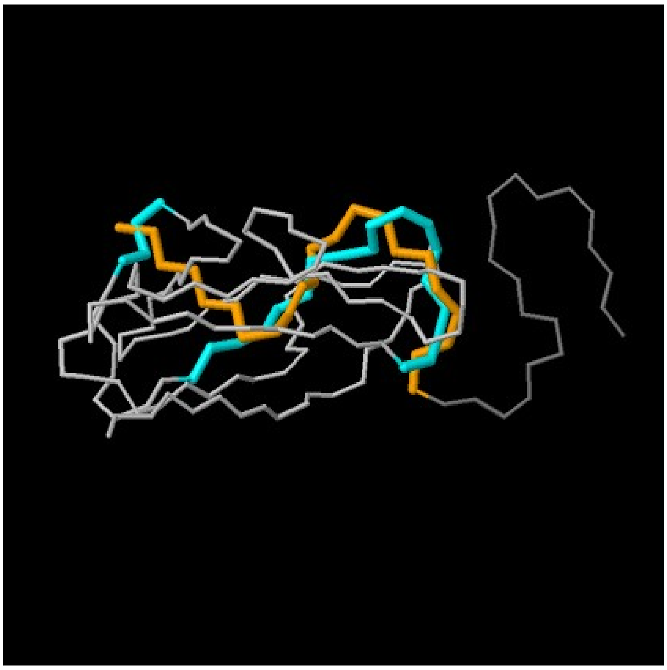

```
Align 2NAO.A.pdb Length1: 42 with 3DOS.C.pdb Length2: 135

P-value: 9.60e-01

Equ: 22

RMSD: 4.29

Score: 23.19

Align-len: 30

Gaps: 8 (26.67%)


Identity: 0.00%

Similarity: 10.00%


1:A                     20:A
|           .    |    .    |
DAEF--------RHDSGYEVHHQKLVFFAE
..:...................
VGDDVVLATGSQDFFVRSIGSKGGKLAAGK
| |     .    |    .    |    .
108:C        120:C



| ... Structurally equivalent and identical residues

: ... Structurally equivalent and similar residues
```

```
. ... Structurally equivalent, but not similar residues.


     To calculate the coordinates of chain 2 aligned on chain 1 apply the following tra
nsformation:


     X1 = (-0.707390)*Xorig + ( 0.009306)*Yorig + ( 0.706762)*Zorig + (   13.601000)

     Y1 = (-0.637529)*Xorig + (-0.440174)*Yorig + (-0.632300)*Zorig + (   73.362000)

Z1 = ( 0.305214)*Xorig + (-0.897864)*Yorig + ( 0.317307)*Zorig + (  -33.218000)
```

Structure Alignment Results

AlignmentDetails:Query: (orange/dark grey) **Subject:** (cyan/light grey)

		Query			Subject	
		Beta-amyloid protein 42			***F1 capsule antigen***	
P-value:9.80e-01	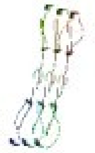	PDB ID:	2NAO	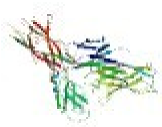	PDB ID:	1P5U
Score:24.91		Chain ID:	A		Chain ID:	C
RMSD:6.32		Length:	42		Length:	130
%Id:10.5%		Similarity:	45%		Similarity:	15%

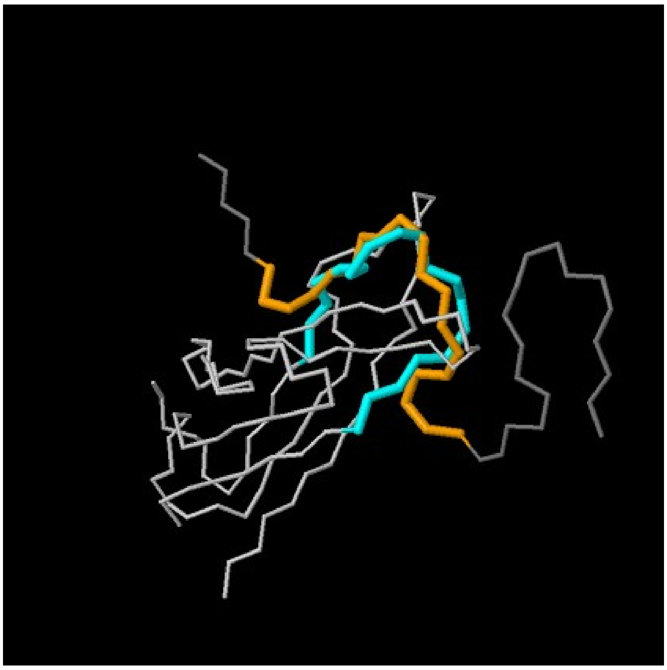

```
Align 2NAO.A.pdb Length1: 42 with 1P5U.C.pdb Length2: 130

P-value: 9.80e-01

Equ: 19
```

```
RMSD: 6.32

Score: 24.91

Align-len: 19

Gaps: 0 (0.00%)


Identity: 10.53%

Similarity: 15.79%


6:A           20:A
|   |    .    |
HDSGYEVHHQKLVFFAEDV
..|..............|.
VRSIGSKGGKLAAGKYTDA
|      |    .    |
123:C            140:C




| ... Structurally equivalent and identical residues

: ... Structurally equivalent and similar residues

. ... Structurally equivalent, but not similar residues.


     To calculate the coordinates of chain 2 aligned on chain 1 apply the following tra
nsformation:


     X1 = (-0.574353)*Xorig + (-0.682623)*Yorig + ( 0.451824)*Zorig + (   29.898000)

     Y1 = ( 0.631490)*Xorig + (-0.720681)*Yorig + (-0.286075)*Zorig + (   40.508000)

Z1 = ( 0.520902)*Xorig + ( 0.121014)*Yorig + ( 0.844995)*Zorig + (  -60.018000)
```

S3 Bioinformatics analysis of 12III1 phage clone

S3.1 Sequence alignment 12III1 pepide-Aβ42-Caf1 by Clustal X2.1

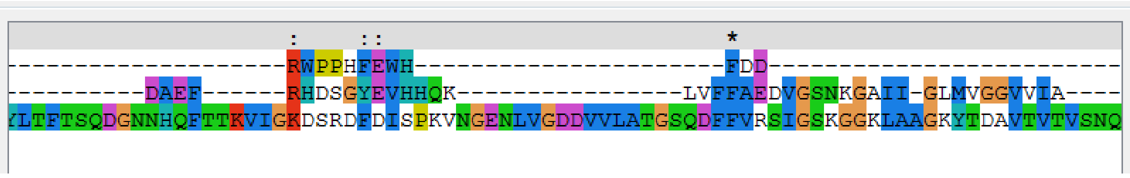

S3.2 PepSurf and Mamitope epitope mappingof 12III1 onto Aβ42 and F1 capsular antigen

PepSurf analysis results of 12III1 exposed peptidewith Aβ42 (PDB ID 2nao chain A, library type : random _AA):

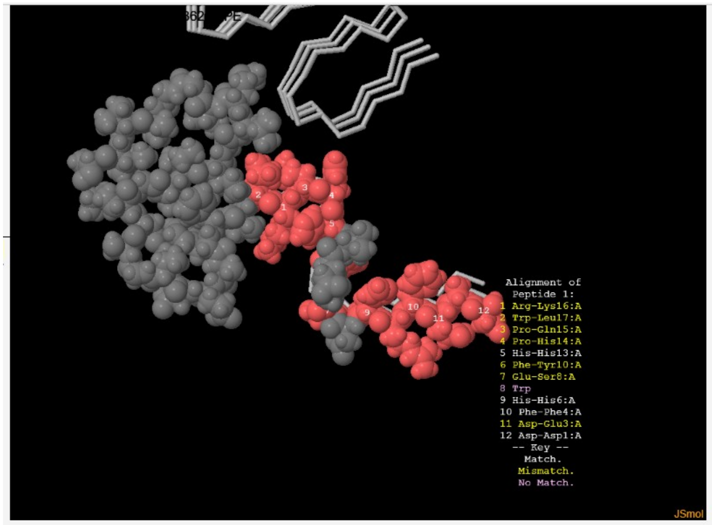


```
*Alignment with highest score*
Alignment score: 656.675 P-value:
0.00244419

peptide: RWPPHFEWHFDD
path:    K16A L17A Q15A H14A H13A
Y10A S8A H6A F4A E3A D1A

Alignment:
RWPPHFEWHFDD
KLQHHYS-HFED
```

Mamitope analysis results of 12III1 (exposed peptide with flanking pVIII sequence) with Aβ42 (PDB ID 2nao chain A, library type : random _AA):

Running Parameters:
Epitope Mapping Algorithm: MAPITOPE
PDB ID: 2nao
Chain identifier: A
Library Type: RANDOM_AA
Stop Codon Modification: TAG = GLN
The Distance Threshold value is: 9.0
The Statistical Threshold value is: 3.0
The Maximum gap "fill-in" is: 3

Best cluster   20-FAEDVG-25
Residues number: 4

PHE20:A
ALA21:A
GLU22:A
ASP23:A
VAL24:A
GLY25:A

Cluster rank: 2 3-EFRH-6
Residues number: 3

GLU3:A
PHE4:A
ARG5:A
HIS6:A

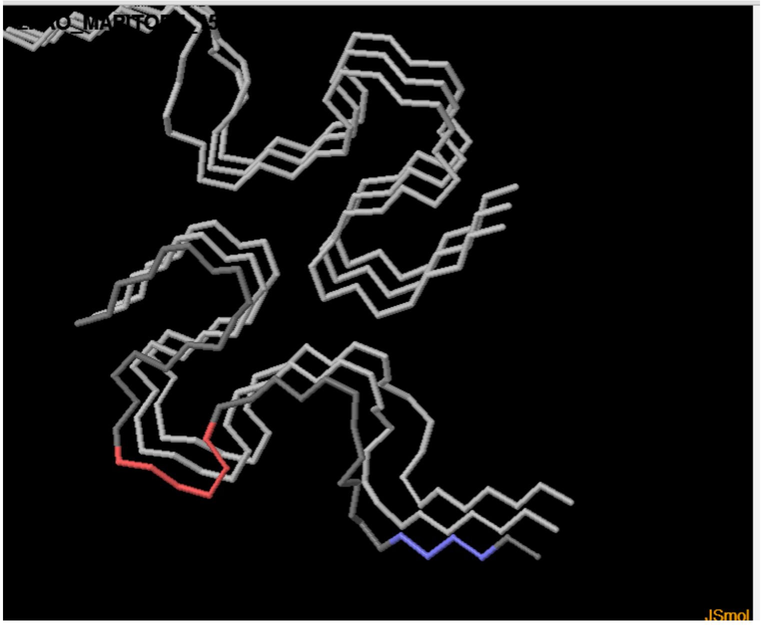

Mamitope and PepSurf combined analysis results of 12III1 (exposed peptide with flanking pVIII sequence with Aβ42 (PDB ID 2nao chain A, library type : random _AA):

Cluster rank: 1 3-EFRH-6
Residues number: 4
intersects cluster number 1 of PepSurf with cluster number 1 of Mamitope

ARG5:A
GLU3:A
PHE4:A
HIS6:A

PepSurf analysis results of 12III1 exposed peptidewithF1 capsular antigen (PDB ID chain C, library type : random _AA):

F1 (1p5u.C)

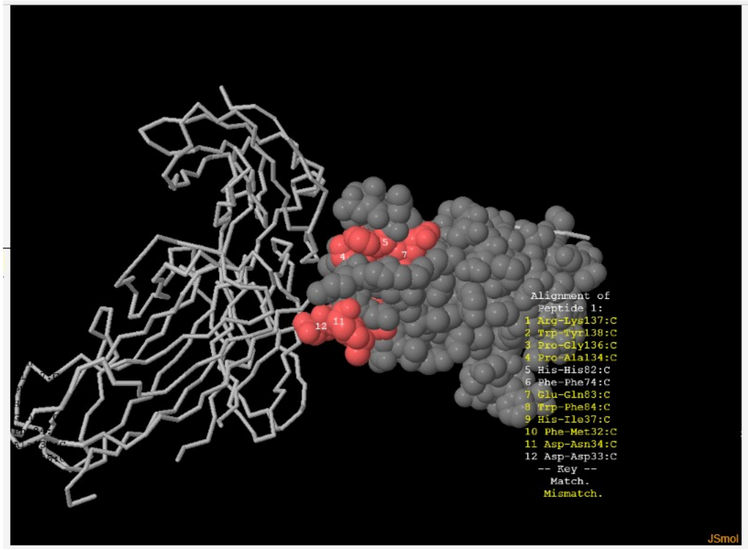

Cluster rank: 2
Residues number: 3

GLY49:C
ASP120:C
PHE122:C

Mamitope analysis results of 12III1 (exposed peptide with flanking pVIII sequence) withF1 (PDB ID 1p5u chain C, library type : random _AA):

Best cluster
Residues number: 4

PHE74:C
HIS82:C
GLN83:C
PHE84:C
ASP140:C

74-F 82-HQF-84 D-140

F1 (3dsn.C)

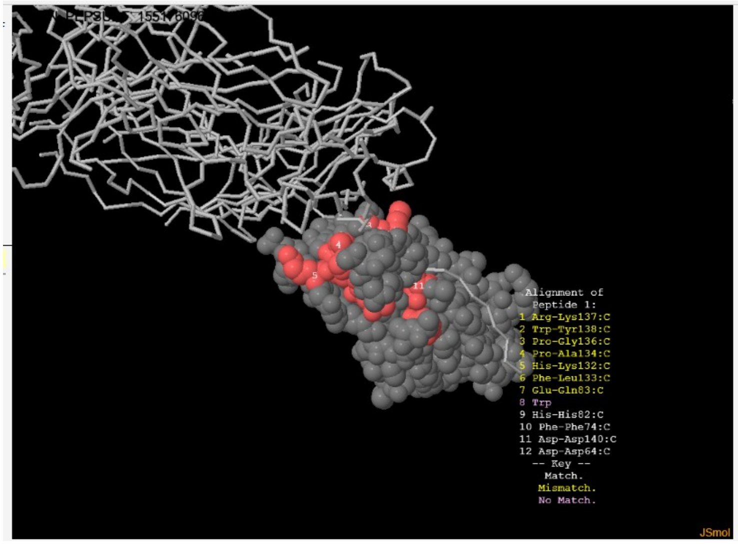

Cluster rank: 3
Residues number: 3

GLY49:C
ASP120:C
PHE122:C

Mamitope analysis results of 12III1 (exposed peptide with flanking pVIII sequence) withF1 (PDB ID 3dsn chain C, library type : random _AA):

Best Cluster
Residues number: 7

ASP92:C
SER93:C
ARG94:C
ASP95:C   92-DSRDFD-97   109-GDD-111
PHE96:C
ASP97:C
GLY109:C
ASP110:C
ASP111:C

Cluster rank: 2
Residues number: 4

PHE74:C
HIS82:C
GLN83:C   74-F   82-HQF-84   D-140
PHE84:C
ASP140:C

F1 (3dpb.C)

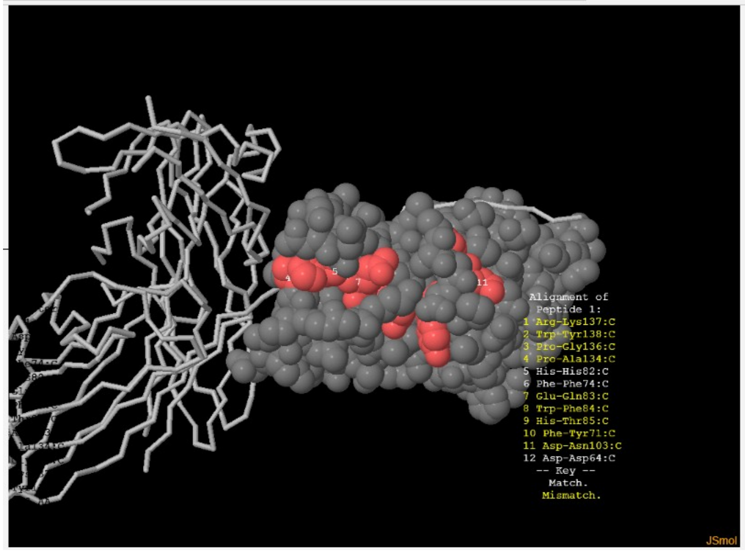

Cluster rank: 2
Residues number: 3

GLY49:C
ASP120:C
PHE122:C

Mamitope analysis results of 12III1 (exposed peptide with flanking pVIII sequence) withF1 (PDB ID 3dpbchain C, library type : random _AA):

Best cluster
Residues number: 4

PHE74:C
HIS82:C
GLN83:C
PHE84:C
ASP140:C

74-F 82-HQF-84 D-140

F1(1z9.C)

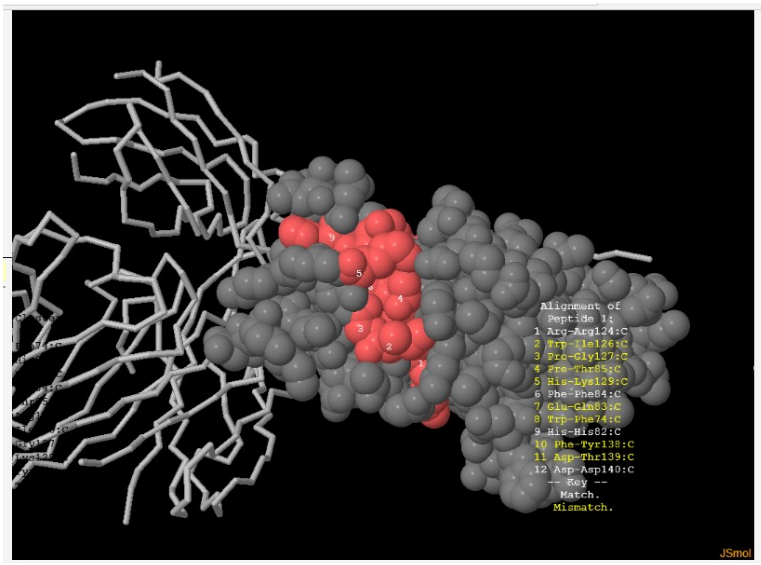

Cluster rank: 2
Residues number: 3

GLY49:C
ASP120:C
PHE122:C

Mamitope analysis results of 12III1 (exposed peptide with flanking pVIII sequence) withF1 (PDB ID 1z9 chain C, library type : random _AA):

Best cluster
Residues number: 4

PHE74:C
HIS82:C
GLN83:C
PHE84:C
ASP140:C

74-F 82-HQF-84 D-140

F1(3dos.C)

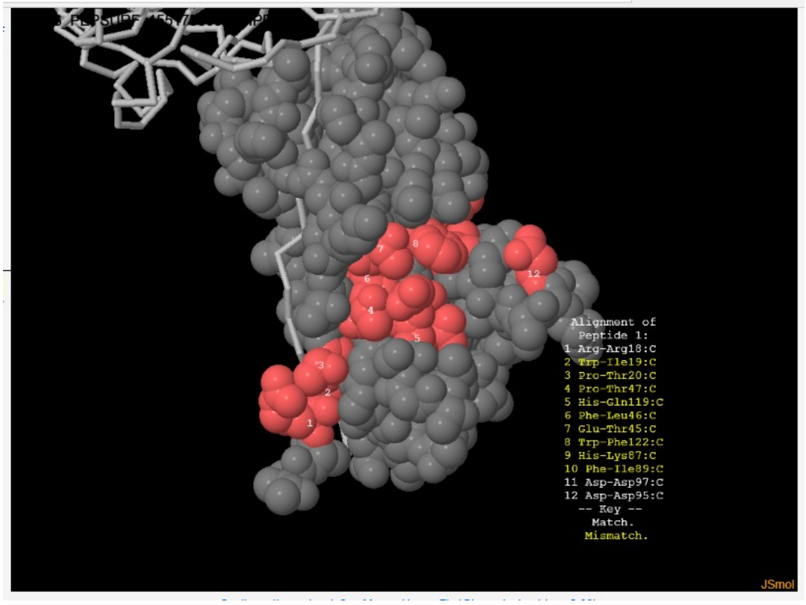

Cluster rank: 3
Residues number: 3

GLY49:C
ASP120:C
PHE122:C

Mamitope analysis results of 12III1 (exposed peptide with flanking pVIII sequence) withF1 (PDB ID 3dos chain C, library type : random _AA):

Best cluster
Residues number: 7

ASP92:C
SER93:C
ARG94:C
ASP95:C 92-DSRDFD-97 109-GDD-111
PHE96:C
ASP97:C
GLY109:C
ASP110:C
ASP111:C

Cluster rank: 2
Residues number: 4

PHE74:C
HIS82:C
GLN83:C 74-F 82-HQF-84 D-140
PHE84:C
ASP140:C

S3.3 Molecular docking prediction of 12III1 phage clone displayed peptide with Aß fibrils

The following imagines show 3D structural modeled interaction between engineered 12III1-pVIII protein in wireframe style with displayed amino acid side positions and beta amyloid fibril in electrostatic surface style (A) and in backbone style coupled to energy map (B). Colors legend of electrostatic and steric clouds: i) Steric favorable non-polar amino acids are included in green regions; ii) Electrostatic favorable, red regions correspond to a nearby negative electro-static charge of amino acids, blue regions correspond to a nearby positive charge of amino acids; iii) Hydrogen donor favorable are yellow; iv) Hydrogen acceptor favorable are light blue.

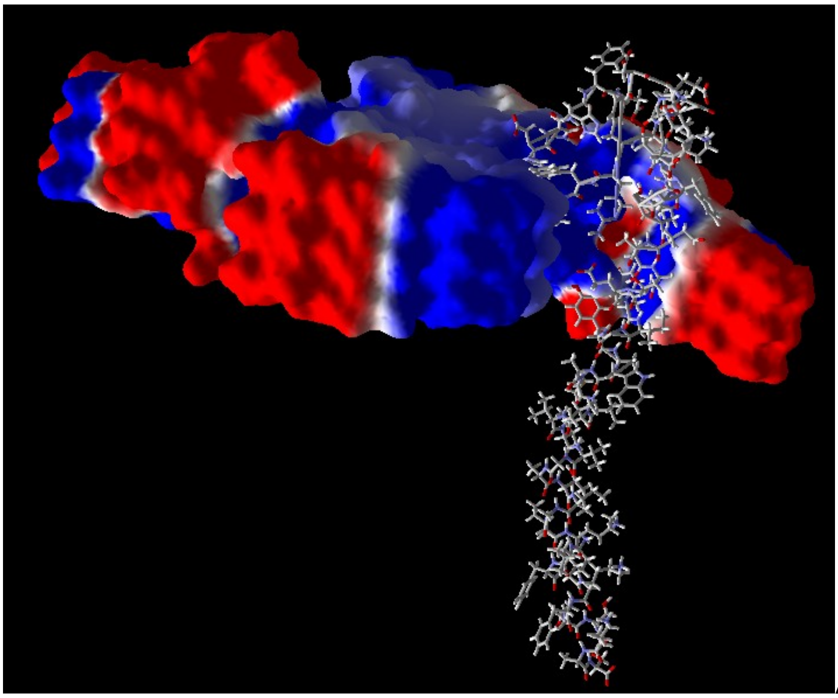
A

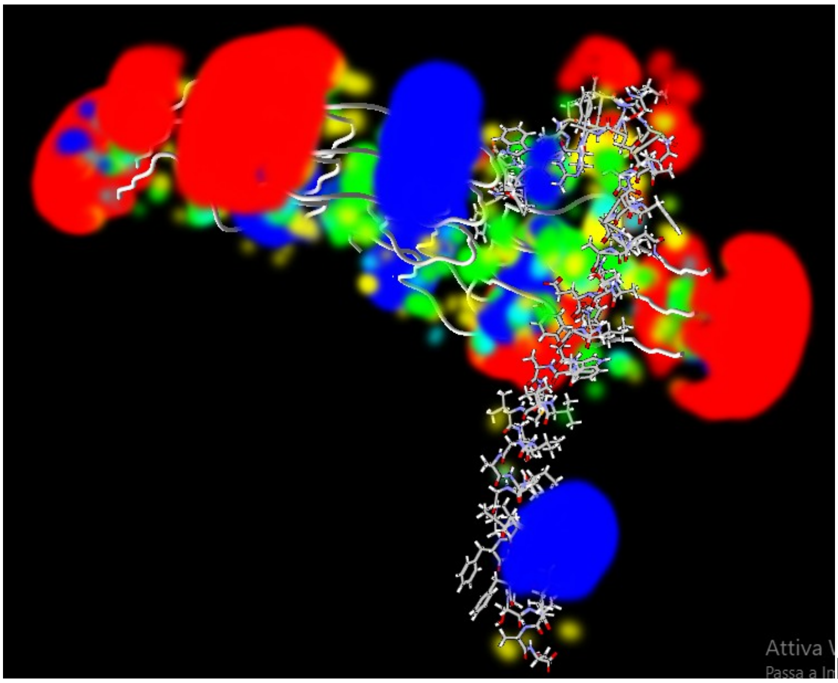
B

The amino acids involved in the interaction are listed in the following tables.

Table S2.12III1-pVIII amino acid residues involved in the interaction with beta amyloid fibril chains.

Beta amyloid fibril chains	Amino acids of 12III1-pVIIIinvolved in the interaction	
	Steric interaction	Electrostatic interaction
Chain A	none	none
Chain B	Phe (11)*	none
Chain C	Trp (7)*, Pro(8)*, Pro (9), His (10)*, Phe (11)*	none
Chain D	Tyr (35), Ile (36), Tyr (38)	Glu (34)
Chain E	Phe (11)*, Ala (32)	none
Chain F	Pro (9)*, His (10)*, Phe (11)*, Glu (12)*, Trp (13)*, Ala (24), Phe (25), Leu (28), Gln (29)	His (10)*, Glu (12)*

*amino acid belonging tothe engineered peptide displayed in 12III1-pVIII
none= no interaction

Table S3. Amino acid residues of beta amyloid fibril chains involved in the interaction with 12III1-pVIII.

	Amino acids of beta amyloid fibril chains involved in the interaction					
	Chain A	Chain B	Chain C	Chain D	Chain E	Chain F
H-bound	none	none	Gly (37), Gly (38)	none	none	His (14), Gln (15)
Stearic interaction	none	Met (35), Val (36), Gly (37)	Met (35), Gly(37), Gly (38),Val (39), Val (40)	Phe (4), Glu (11), Val (12), His (13), His (14)	Phe (4), His (14)	Phe (4), His (6), His (14), Gln (15), tyr (10), Lys (16), Leu (17)
Electrostatic interaction	none	none	none	His (14)	none	none

none= no interaction
